# Exogenous antibiotic resistance gene contributes to intestinal inflammation by modulating the gut microbiome and inflammatory cytokine responses in mouse

**DOI:** 10.1080/19490976.2022.2156764

**Published:** 2022-12-27

**Authors:** Rong Tan, Min Jin, Zhengshan Chen, Yifan Shao, Yuanyuan Song, Jing Yin, Lifang Wang, Tianjiao Chen, Junwen Li, Dong Yang

**Affiliations:** Department of Environment and Health, Tianjin Institute of Environmental and Operational Medicine, Key Laboratory of Risk Assessment and Control for Environment & Food Safety, Tianjin, China

**Keywords:** eARGs, gut microbiome assembly, inflammatory cytokine response, metabolic profiles, intestinal inflammation

## Abstract

Dysregulation of the gut microbiota by environmental factors is associated with a variety of autoimmune and immune-mediated diseases. In addition, naturally-occurring extracellular antibiotic resistance genes (eARGs) might directly enter the gut via the food chain. However, following gut microbiota exposure to eARGs, the ecological processes shaping the microbiota community assembly, as well as the interplay between the microbiota composition, metabolic function, and the immune responses, are not well understood. Increasing focus on the One Health approach has led to an urgent need to investigate the direct health damage caused by eARGs. Herein, we reveal the significant influence of eARGs on microbiota communities, strongly driven by stochastic processes. How eARGs-stimulate variations in the composition and metabolomic function of the gut microbiota led to cytokine responses in mice of different age and sex were investigated. The results revealed that cytokines were significantly associated with immunomodulatory microbes, metabolites, and ARGs biomarkers. Cytokine production was associated with specific metabolic pathways (arachidonic acid and tryptophan metabolic pathways), as confirmed by *ex vivo* cytokine responses and recovery experiments *in vivo*. Furthermore, the gut microbial profile could be applied to accurately predict the degree of intestinal inflammation ascribed to the eARGs (area under the curve = 0.9616). The present study provided a comprehensive understanding of the influence of an eARGs on immune responses and intestinal barrier damage, shedding light on the interplay between eARGs, microbial, metabolites, and the gut antibiotic resistome in modulating the human immune system.

## Graphical Abstract

**Figure:**
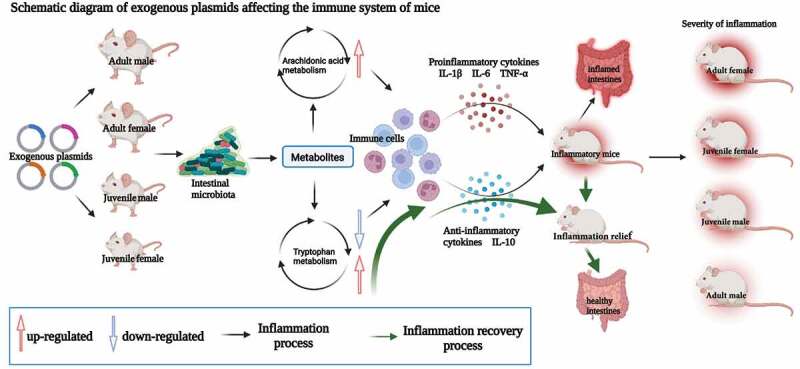

## 1. Introduction

The human gut microbiota is densely colonized by micro-organisms, which have dynamic interactions with each other and with the host.^1^ The gut microbiota is considered to be one of the crucial factors contributing to human health, which benefits host health through metabolic cross-feeding and by triggering the immune system;^2^ however, gut microbiome dysbiosis can have pernicious effects. Currently, dysregulation of the gut microbiota is associated with various autoimmune and immune-mediated diseases, including: diabetes, rheumatoid arthritis, multiple sclerosis, systemic lupus erythematosus, inflammatory bowel diseases (IBDs), and atherosclerosis.^3,4^

The age and sex of the host are important factors affecting the stability of the intestinal microbiota,^5^ and the gut microbial community shifts throughout the host’s life span. There is increasing evidence suggesting a correlation between the gut microbiota and age or biological sex. The species and relative abundance of *Bacteroide*s increases with age, while *Actinobacteria* (including 15 Bifidobacterium species) decreases with age.^6^ In addition, the gut microbiota presents a strong positive association between age and alpha diversity in young adults (< 40 years old), and women have a higher alpha diversity than men.^7^ Several studies have shown that sex differences in the gut microbiota appear soon after entering puberty,^8^ which are driven in part by sex hormones, and the microbiome alteration contributes to the sexual dimorphism of differences in susceptibility to autoimmune and infectious diseases.^9^ Although the gut microbiome and the host immune system will continue to shape each other throughout the life cycle,^10^ increasing evidence suggests that the differences in the nature and strength of immune responses between men and women lead to sex-specific differences in the prevalence, manifestations, and outcomes of infectious diseases, autoimmune diseases, and even malignancies, which might be ascribed to the gut microbiome.^9^ Thus, it is key to consider the impact of exogenous factors that have a significant potential to jeopardize public health via the gut microbiota of hosts of different age and sex.

Over the years, antibiotic resistance has been regarded as one of the major threats to global human health. These concerns have increased with the discovery and rapid spread of antibiotic resistance genes such as *bla_NDM_,bla_kpc_, mcr-1, PMQR*, and *optrA*. Furthermore, eARGs as a common form of ARGs in the natural environment, have been detected in surface water and effluents of wastewater treatment plants, and even in tap water,^11^ vegetables, and beef.^12^ Therefore, eARGs might directly enter the human intestine through drinking water and food, and then interact with the intestinal microbiota. Many studies have shown that ARGs can transfer into the intestinal microbiota.^13,14^ However, the ecological processes shaping the microbiota community upon eARGs entry into the intestines are not well understood. Therefore, a study of the influence of eARGs on the gut microbiome would provide new insights into microbiota ecology.

The gut microbiota regulates multiple aspects of microbial metabolite pools, which can affect the intestinal barrier and the polarization of immune cells. Therefore, many disease are associated with a dysregulated gut microbiome composition, metabolite disturbances, and active inflammation.^3^ IBDs are inflammatory disorders of the intestine, which are associated with a disrupted gut microbial composition, as well as active inflammation and metabolite disturbances, including alterations to short chain fatty acids (SCFAs) and bile acid pathways.^15^ The increased abundance of several microbial species, such as *Parabacteroides sp., Dialister invisus*, or *Lachnospiraceae*, and certain metabolites, such as tryptophan metabolites, are involved in celiac disease in infants.^16^ In addition, most signaling metabolites, including polysaccharides, sphingolipids, and muropeptides, which are produced by large numbers of different gut bacteria, can evade and modulate the immune system.^3^ However, when eARGs enter the intestines, the interplay among the microbiota composition, metabolic function, and immune responses is not clear.

Herein, to better understand how eARGs influence the microbiota composition, metabolic function, immune responses, and the host health status, mice of different age and sex were administered with a plasmid harboring the colistin resistance gene, *mcr-1*, which has shown rapid spread into seven pathogenic species in more than 30 countries. We used a neutral community model to disentangle the influences of the eARGs on the gut microbiota assembly. Finally, the associations among the microbiota, intestinal metabolites, serum inflammatory cytokines, serum metabolites, the gut antibiotic resistome, and *ex vivo* blood stimulations with four microbial stimuli were comprehensively analyzed. In addition, a machine learning method was used to predicting indicators of intestinal injury, which could provide direct evidence for the health impact of eARGs. The aim of the study was to reveal the interplay between eARGs, microbes, metabolites, and the gut antibiotic resistome in immune system regulation. The present study lays the groundwork for increasing our understanding of the impact of eARGs on specific hosts (females), and provides directions to develop therapeutic strategies or dietary interventions.

## 2. Results

### 2.1 Effects of eARGs on body weight and serum immune cytokines in mice

**Figure 1.**
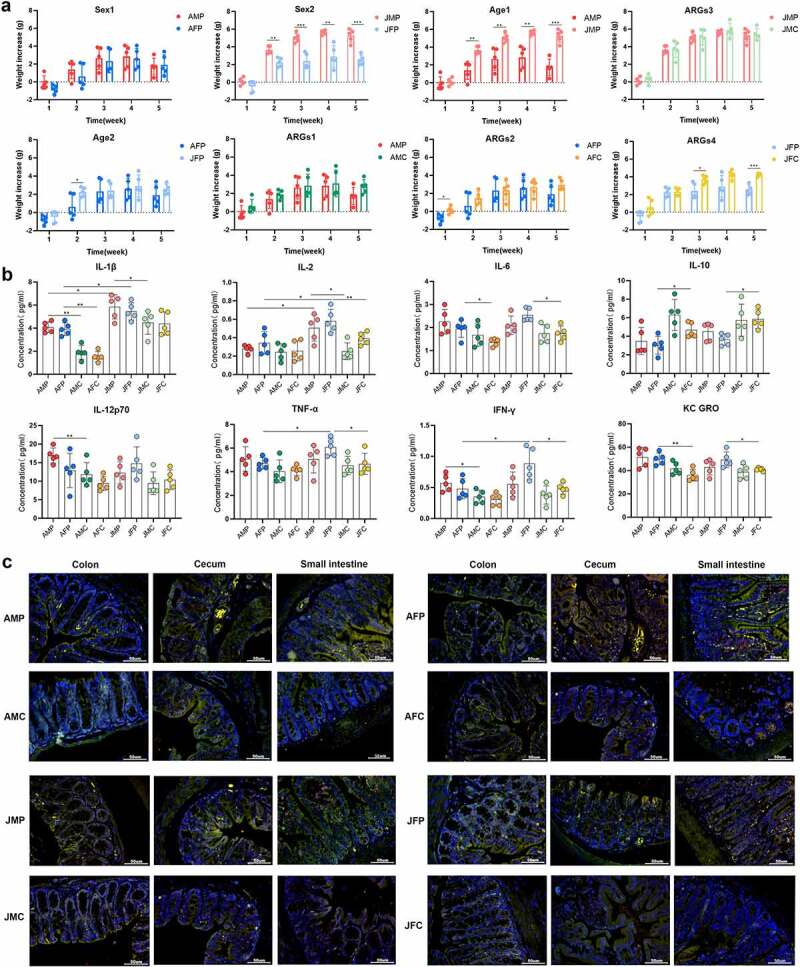
Effects of eARGs on the growth and immune system of mice. Body weight change of mice in the different age and sex groups for 1–5 weeks (a). Serum cytokine concentrations in mice in the different age and sex groups (b). Immunofluorescence staining of the cecum, colon, and small intestine sections of mice of different ages and sexes, the yellow dot indicates the cells after the superposition of CD3 and CD4, namely inflammatory cells. (c). To facilitate the identification of each group in the figure, the adult male mice treated with the plasmid are abbreviated as the AMP group, the adult male control group is termed AMC, the adult female mice treated with the plasmid are abbreviated as the AFP group, the adult female control group is termed AFC, the juvenile males treated with the plasmid are designated as the JMP group, the juvenile male control group is termed JMC, the juvenile female mice treated with the plasmid are designated as the JFP group, and the juvenile female control group is termed JFC. Each subsequent figure is marked with these abbreviations.

The changes in body weight in each group indicated that the eARGs had an impact on the increment in mouse body weight. After the eARGs entered the intestines, the body weight increment of the mice was lower in the experimental group than in the control group at most time points, and had a marked impact in the early stage. After eARGs administration, the weight gain of the adult male mice was higher than that of the adult female mice, which was most significant in the first week (p < .05). The body weight gain of juvenile male mice was also significantly higher than that of the juvenile female mice (p < .05), and some of the adult female and juvenile female mice showed decreased body weight in the first and second weeks (Figure 1a), indicating that the eARGs affected the growth of mice, and had a greater impact on female mice.

After exposure to eARGs, the levels of inflammatory cytokines, including interleukin-1β (IL-1β), IL-2, IL-6, IL-12p70, tumor necrosis factor-α (TNF-α), interferon-γ (INF- γ), and chemokine (Keratinocyte chemoattractant (KC)/human growth-regulated oncogene (GRO)), were higher in the experimental groups than in the control groups. Among them, the IL-1β levels in the experimental group of adult males, adult females, and juvenile males were significantly higher than that in the control group (p < .05). IL-2 levels in the experimental group of juvenile males and females were significantly higher than that in the control group (p < .05). The levels of IL-6 and KC/GRO in the experimental group of adult and juvenile females were significantly increased compared with those in the control group (p < .05). The IL-12p70 levels in the experimental group of adult male mice was significantly higher than those in the control group (p < .05). TNF-α levels in the experimental group of juvenile females were significantly higher than those in the control group (p < .05). The level of IFN- γ in the experimental group of adult male and juvenile females was significantly higher than that in the control group (p < .05). The levels of IL-10 in each group were lower than those in the control group, and the levels of IL-10 in the experimental group of adult females and juvenile females were significantly lower than those in the control group (p < .05; Figure 1b). These results showed that eARGs treatment caused immune dysfunction, particularly in female mice.

Furthermore, pathological observation of each intestinal segment of mice was carried out using immunofluorescence staining, which showed that the structural changes to the cecum were the most severe in the experimental group, followed by the colon and small intestine (Figure 1c). Following exposure to eARGs, we observed that in the experimental group, the overall structure of cecum tissue was slightly abnormal, the mucosal layer appeared abnormal, part of mucosal epithelium had fallen off, and inflammatory cell aggregation was seen in the mucosal layer. Among the groups, the mucosa of adult female mice was obviously infiltrated by inflammatory cells compared with the other three groups. After adding the eARGs, the colon tissue in the experimental group showed a slightly abnormal overall structure, goblet cells and inflammatory cells were gathered, and the submucosa showed slight edema. In addition, the overall structure of adult female small intestine in the experimental group was disturbed, and the mucosal layer showed marked infiltration by inflammatory cells. The structure of the other three groups was basically normal, although a few inflammatory cells could be seen (**Fig. S1a; Table S1**). The staining results showed that eARGs treatment affected the intestinal structure and function of mice, causing inflammation. The effect was most significant in adult females.

### 2.2 Effect of eARGs on the intestinal microbiota in mice

**Figure 2.**
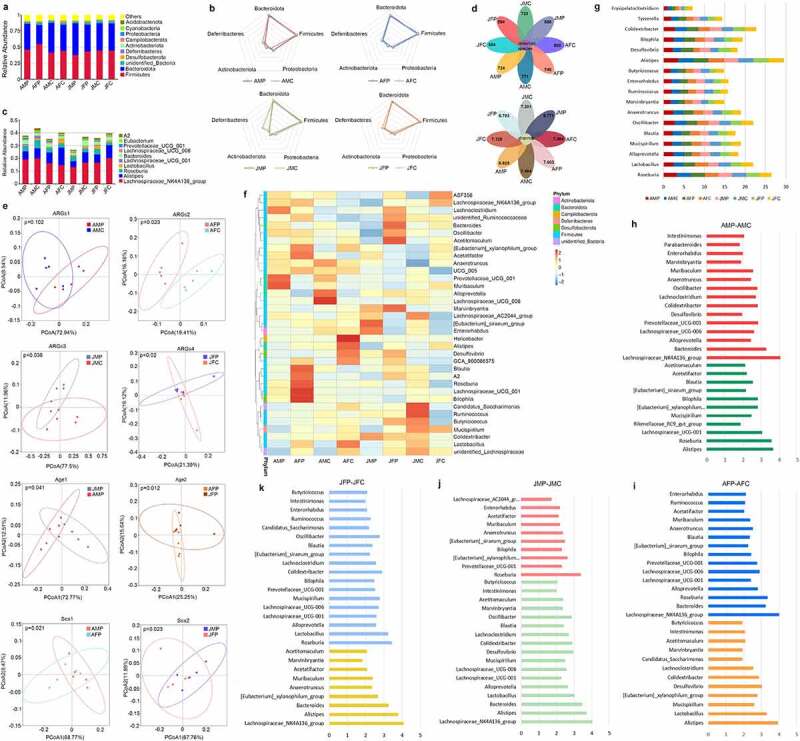
Effects of eARGs on the structure and abundance of the intestinal microbiota in mice. Histogram of the distribution of the top 10 species at the phylum level of mice in the different age and sex groups (a). Radar map of the distribution of the top 5 species at the phylum of mice in same age and sex groups (b). Histogram of the distribution of the top 10 species at the genus of mice in different age and sex groups (c). Petal diagram of observed species and species diversity (Shannon index) of the mice in each group (d). Principal coordinate analysis (PCoA) according to eARG addition or not, age, and sex in each group (e). Heat map of the distribution of species at the genus level and corresponding phylum level of the mice in each group (f). Histogram of the distribution of probiotics and pathogens of mice in the different age and sex groups (g). Histogram of the distribution of biomarker bacteria of mice in the same age and sex groups (h, i, j, k) .

Analysis of the intestinal microbiota of each group showed that at the phylum level, *Bacteroidetes* decreased (46.52% to 40.31%) and *Firmicutes* increased (41.49% to 46.15%) after exposure to eARGs in the adult male group. Similarly, *Bacteroidetes* decreased (40.65% to 29.86%) and *Firmicutes* increased (44.36% to 54.72%) in the adult female group. *Bacteroidetes* increased (42.64% to 49.62%) and *Firmicutes* decreased (44.84% to 37.77%) in the juvenile female group, while decreases were observed for both *Bacteroidetes* (42.50% to 41.97%) and *Firmicutes* (44.64% to 43.23%) in the juvenile male group. (Figure 2a). In addition, following exposure to eARGs, *Proteobacteria* of the other three groups increased significantly, and the *Deferribacteresxian* decreased significantly, except in the juvenile male group (Figure 2b). At the genus level, in the mice exposed to eARGs, the adult male mice had higher levels of *lachnospiraceae_NK4A136* (19.26%), *Alistipes* (6.39%), *Roseburia* (4.19%), *Bacteroides* (3.18%); the adult female mice had higher levels of *Lachnospiraceae_NK4A136* (19.99%), *Alistipes* (7.99%), *Roseburia* (6.39%), *Bacteroides* (2.13%), *Lachnospiraceae_UCG-001* (2.02%), *Lactobacillus* (1.37%), and *Colidextribacter* (1.46%); the juvenile male mice had higher levels of *Lachnospiraceae_NK4A136* (13.25%), *Alistipes* (4.72%), *Roseburia* (3.88%), and *Bacteroides* (1.60%); and the juvenile female mice had higher levels of *Lachnospiraceae_ NK4A136* (16.73%), *Alistipes* (8.22%), *Bacteroides* (4.26%), *Roseburia* (2.94%), and *Lactobacillus* (1.64%) (Figure 2c). The results of the microbiota distribution showed that treatment with eARGs significantly affected the structure of the intestinal microbiota, which was manifested in decrease levels of *Bacteroidetes* and increased levels of *Firmicutes* and *Proteobacteria*.

According to the diversity analysis of the samples in each group, compared with control groups, the observed species in the experimental groups of each age and sex exposed to eARGs were significantly lower. Compared with that in the control group, the Shannon index was significantly lower in the experimental group of each age and sex (Figure 2d). Thus, the diversity and species richness of mice in all age and sex groups were significantly reduced after exposure to eARGs, indicating that the composition and distribution of original microbiota was disturbed by the eARGs. Based on age and sex, principal coordinate analysis (PCoA) indicated that there was no significant difference between the adult male experimental group and the control group (p = .102). The other three groups showed significant differences between the experimental group and the control group. The difference among juvenile females was the most significant (p = .02), followed by adult females (p = .023) and juvenile males (p = .036). Thus, treatment with eARGs had a greater effect on the intestinal microbiota of female mice (Figure 2e). The results showed that exposure to an eARG could significantly change the distribution of the gut microbiota and the types and abundance of specific species, which might increase pathognomonic metabolites and metabolic pathways, ultimately affecting immune responses.

The specific analysis of the different microbiotas shows that most of the top 35 bacteria are distributed in the *Firmicutes*, and there were significant differences in the microbiota distribution among the groups (Figure 2f). Compared with that in the control groups, probiotics, including *Lactobacillus, Oscillibacter, Roseburia*, and *Butyricicoccus*, which can decompose carbohydrates; and *Alloprevotella, Mucispirillum, Blautia, Anaerotruncus, Marvinbryantia, Ruminococcus*, and *Enterorhabdus*, which are mainly responsible for fermentation in the intestine, significantly decreased after exposure to eARGs. The levels of certain pathogenic bacteria, such as *Desulfovibrio, Alistipes, Bilophila, Colidextribacter, Tyzzerella*, and *Erysipelatoclostridium* were significantly higher compared with those in control groups (Figure 2g). Furthermore, after exposure to eARGs, the bacterial biomarkers of each experimental group were altered (Figure 2h-2k**, Table S2**), suggesting that eARGs contributed to significant changes in the gut microbiota and reshaped the bacterial biomarkers in the mouse intestines.

### 2.3 Effect of eARGs on the intestinal microbiota community assembly in mice

**Figure 3.**
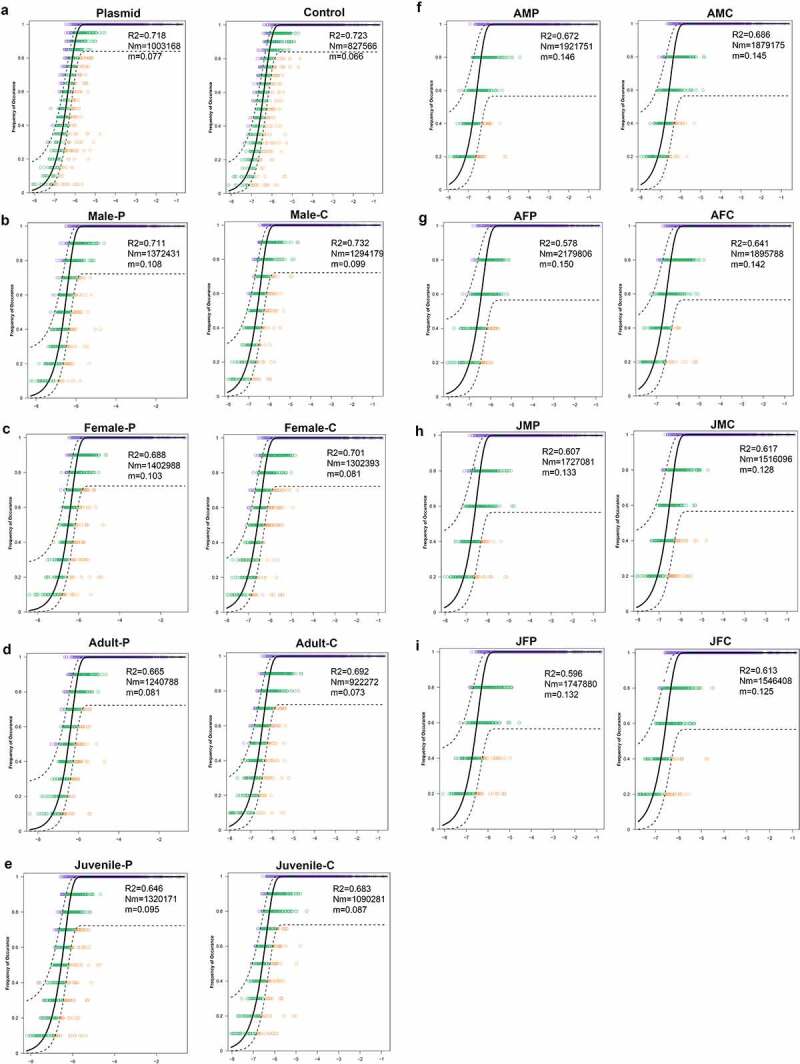
Effects of eARGs on the intestinal microbiota community assembly. The neutral community model of mice with exogenous plasmids (a, b), different age and sexes (c-e), and different age and sex added with exogenous plasmids (f-i). Among them, R^2^ refers to the influence rate of environmental factors on the community. The closer R^2^ is to 1, the less the community is affected by the environment. Nm refers to the probability that the random loss of individuals in a community is replaced by the dispersion of meta-communities rather than reproduction in local communities. A higher Nm value indicates a higher level of migration, and m indicates mobility. The greater the value of m, the greater the mobility. The black solid line indicates the most fitted value of the model in the medium-sized community, and the dotted line represents the 95% confidence interval of the model. A sample with a frequency higher or lower than that predicted by the neutral community model is displayed in different colors.

To clarify the potential impact of eARGs on the assembly of the gut microbial community, the neutral community model (NCM) was used to predict the relationship between the occurrence frequency and relative abundance of operational taxonomic units (OTUs) in the sub community data sets. The results showed that the NCM successfully estimated most of the relationships between the occurrence frequency and its relative abundance alteration, and had a high interpretation rate before and after eARGs exposure (R^2^ > 0.7; Figure 3a). This indicated that the stochastic process is very important for the formation of the mouse intestinal microbial community assembly; however, the explained variation of NCM tended to decline in the experimental groups, indicating that the eARGs primarily shifted the assembly of the bacterial community. More importantly, compared with each control group, the relative abundance variations were reduced more significant in the experimental groups, indicating that eARGs had greater influence on the assembly of microbiota community in female mice, as well as the juvenile mice.

Concordant results were obtained at the age or sex levels. The NCMs also largely explained the community variance before and after eARGs exposure (R^2^ > 0.6; Figure 3b-e). The age and sex of the mice affected the assembly of the microbial communities, and among them, females had lower randomness and a higher diffusion rate than males before and after eARGs exposure. In addition, there was a similar assembly mechanism in juvenile and adult mice, indicating that the intestinal community stability of female and juvenile mice was lower than that of male and adults; therefore, their assembly is more vulnerable to eARGs. Interestingly, the influence of eARGs on the assembly of the microbial communities was higher in juvenile and female than that in adult and male mice (Figure 3b-e).

The Nm-value was higher for the gut microbial taxa in the experiment group (Nm = 1003 and 168, male/female) than in the control group (Nm = 827 and 566, male/female), and the m value was estimated as 0.077 in the experiment group but 0.066 in the control group (Figure 3a). Thus, eARGs exposure increased the species dispersal ability of the gut microbial community (Figure 3h-i). The results of comparing the potential effects of eARGs on the species dispersal ability of gut microbial community in mice showed it had the greatest impact on adult females (Figure 3g), followed by juvenile females (Figure 3i), and had least effects on juvenile males and adult males (Figure 3f, h), indicating that the species dispersal ability of the gut microbiota was significantly affected by eARGs, and the adult female mice were most vulnerable to eARGs.

### 2.4 Effect of the eARGs on the gut antibiotic resistome in mice

**Figure 4.**
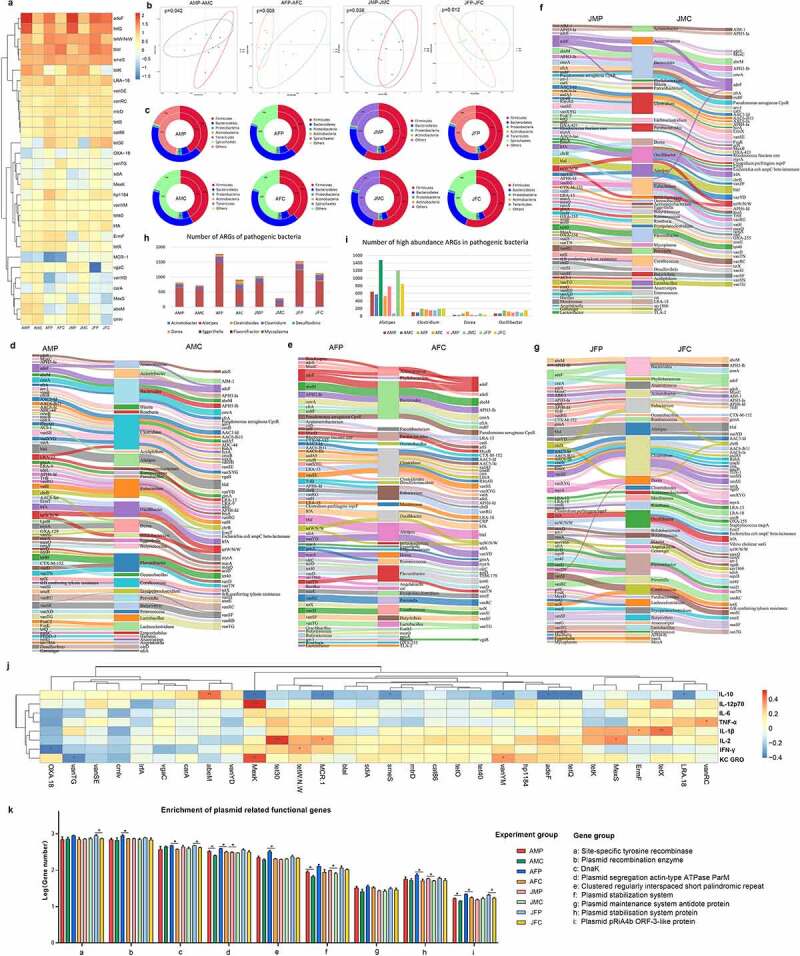
Effect of eARGs on the intestinal microbiota antibiotic resistome. Heat map of the distribution of the top 30 intestinal ARGs of mice in different age and sex groups (a). Principal component analysis (PCA) of the intestinal ARGs of mice in the different age and sex groups (b). Double circle diagram of the species distribution of ARGs at the phylum level in each group. The inner circle shows the distribution of host bacteria of ARGs, and the outer circle shows the distribution of intestinal microbiota (c). Sanggi diagram of ARGs and their host distribution at the genus level of mice in different age and sex groups (d-g). Histogram of pathogenic bacteria distributed by ARGs in each group (h). Histogram of pathogenic bacteria with high abundance ARGs distribution (i). Heat map of the correlation between intestinal ARGs and serum cytokines. Among them, red represents positive correlation, blue represents negative correlation, and * represents significance (j). Histogram of gene quantity distribution enriched in eARGs-related pathways in different age and sex groups (k) .

To determine the distribution of the antibiotic resistance ontology (ARO) of mice in all groups, a cluster heat map was drawn using the top 30 selected ARO-related genes. Further analysis of the top 5 ARO-related genes in different age and sex groups showed that the most abundant ARO-related genes in the adult male experimental group and control group, as well as the juvenile male experimental group and control group were *adeF, tetQ, blaI, tetW/N/W* and *tetK*. While the most abundant ARO-related genes in the adult female experimental group and control group, as well as juvenile female experimental group and control group were *adeF, tetW/N/W, blaI, tetQ* and *smeS*, indicating that the intestinal resistance genes of the same sex tend to be more consistent (Figure 4a). Further comparisons showed that eARGs treatment significantly affected the gut antibiotic resistome in different age and sex mice, with the most significant impact on adult and young females (Figure 4b).

The ARGs in each group are distributed in different host bacteria. At the phylum level, they are mainly distributed in the *Firmicutes, Bacteroidetes, Proteobacteria, Actinomycetes, Tenericutes*, and *Spirochetes* (Figure 4c). Analysis of the distribution of ARGs at the genus level in each group showed that *Bacteroides* is the host with the widest distribution of ARGs in adult males and juvenile males, and *Clostridium* is the host with the widest distribution of ARGs in adult females and juvenile females, indicating that after eARGs treatment of mice in different age and sex groups, different bacterial groups are shaped in the intestine to become the host bacteria of ARGs (Figure 4d-4g**, Table S3**). These host bacteria included probiotics, opportunistic pathogens, and symbionts. At the genus level, probiotics harboring ARGs mainly included *Lactobacillus, Oscillibacter, Bifidobacterium, Roseburia, Butyricicoccus, Acidiphilium*, and *Lactonifactor*. Opportunistic pathogens with ARGs included *Alistipes, Prevotella, Clostridium, Erysipelatoclostridium, Acinetobacter, Eisenbergiella*, and *Eggerthella*. Symbiotic bacteria with ARGs included *Bacteroides, Enterococcus, Eubacterium, Desulfovibrio*, and *Dorea*. (Figure 4h, i). It is a concern that when these opportunistic pathogens carry ARGs, they may cause serious health hazards.

In terms of the immune responses following eARGs treatment, some ARGs biomarkers emerged in the gut antibiotic resistome simultaneously. Further analysis of the correlation between high abundance ARGs and inflammatory cytokines showed that *adeF, tetQ, tetW/N/W, tet 30, mcr-1*, and *MexK* correlated positively with inflammatory cytokines (Figure 4j). Interestingly, the analysis of genes enrichment pathways revealed that the following pathways were related to plasmid transfer and diffusion: Site-specific tyrosine recombinase, Plasmid recombination enzyme, Plasmid segregation actin-type ATPase ParM, Clustered regularly interspaced short palindromic repeat, Plasmid stabilization system, Plasmid maintenance system antidote protein, Plasmid stabilization system protein, and Plasmid pRiA4b ORF-3-like protein. Compared with the control groups, the number of genes enriched in these pathways was higher in the experimental groups, and was most significant in the female groups. We revealed that eARGs treatment elevated the expression of relevant regulatory genes to promote the horizontal transfer of plasmids, leading to a health risk (Figure 4k).

### 2.5 Effects of the eARGs on the fecal and serum metabolomes in mice

**Figure 5.**
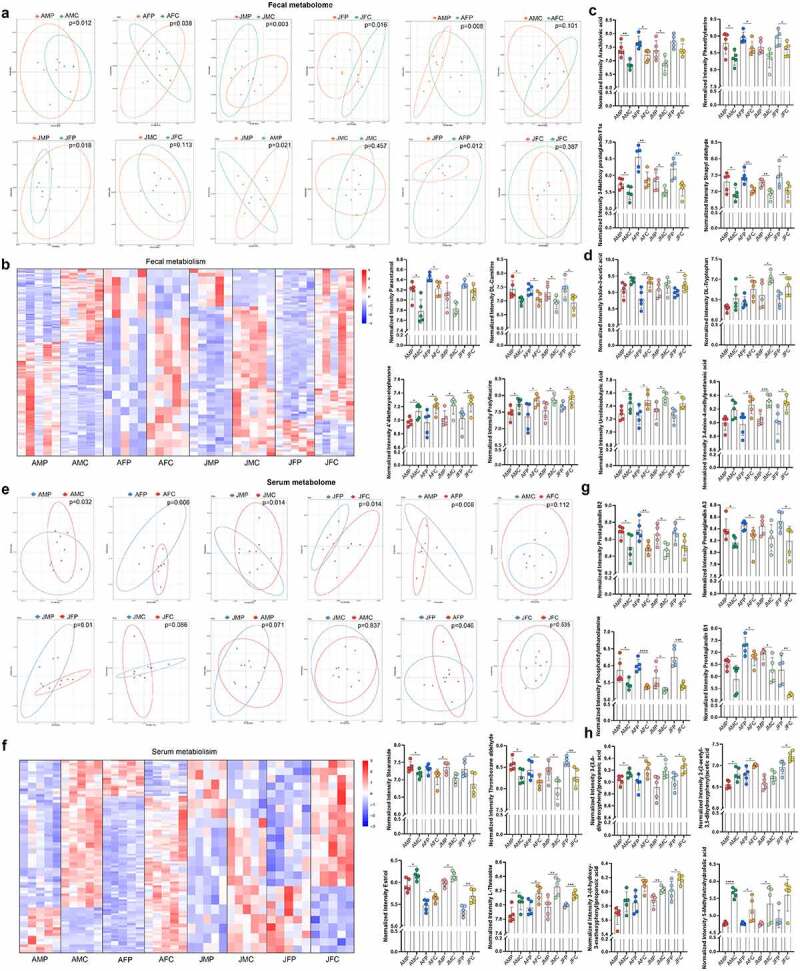
eARGs caused differences in fecal metabolites and serum metabolites in mice. PCA of fecal metabolites of mice in the different age and sex groups (a). Heat map of fecal metabolite distribution of mice in the different age and sex groups (b). Scatter plot with bar of pro-inflammatory metabolites with significant differences in feces of mice in different age and sex groups (c). Scatter plot with bar of anti-inflammatory metabolites with significant differences in feces of mice of different age and sex groups (d). PCA of serum metabolites of mice in the different age and sex groups (e). Heat map of serum metabolites distribution of mice in the different age and sex groups (f). Scatter plot with bar of pro-inflammatory metabolites with significant differences in serum of mice in the different age and sex groups (g). Scatter plot with bar of anti-inflammatory metabolites with significant differences in the serum of mice in different age and sex groups (h) .

The fecal metabolites of each group were analyzed using PCA, which showed that there were significant differences in fecal metabolites in the mice in each experimental group after eARGs treatment compared with those in the control group (p < .05; Figure 5a). Before eARGs treatment, there was no significant difference between adult males and females (p = .101); however, after eARGs treatment, there was a significant difference (p = .008). There was no significant difference between juvenile males and females before eARGs addition (p = .113); however, after eARGs treatment, there was a significant difference (p = .018; Figure 5a). PCA analysis of serum metabolites showed that compared with the control group, there were significant differences in serum metabolites in the mice in each experimental group after eARG treatment (p < .05; Figure 5e). Before eARGs treatment, there was no significant difference between adult males and females (p = .112); however, after eARGs treatment, there was a significant difference (p = .008). There was no significant difference between juvenile males and females before eARGs addition (p = .086); however, after eARGs treatment, there was a significant difference (p = .01; Figure 5e). These results showed that eARGs caused significant changes in the fecal and serum metabolomes in the mice in each group, indicating that eARGs shaped specific metabolites significantly via the gut microbiota in mice in the different age and sex groups.

Cluster analysis of fecal and serum metabolites showed that after eARG treatment, significant differences could be observed in the distribution of metabolites in each experimental group, compared with that in the control groups (Figure 5b, f). The specific metabolites that were higher in the feces of the experimental group compared with those in the control group included Arachidonic acid, Phenethylamine, 3-Methoxy prostaglandin F1α, Sinapyl aldehyde, Paracetamol, and Prostaglandin E2 (p < .05; Figure 5c). The significantly reduced metabolites included Indole-3-acetic acid, DL-Tryptophan, 4’-Methoxyacetophenone, Prolylleucine, Ureidoisobutyric Acid, and 3-Amino-4-methylpentanoic acid (p < .05; Figure 5d). The significantly increased metabolites were mainly concentrated in prostate and aldehydes, and the significantly reduced metabolites were mainly concentrated in SCFAs, ketones, and amino acids. The specific metabolites that were higher in the feces of the experimental group compared with those in the control group included Prostaglandin B2, Thromboxane aldehyde, Prostaglandin A3, Phosphatidylethanolamine, Prostaglandin B1, and Stearamide (p < .05; Figure 5g). The significantly reduced metabolites included 3-(3,4-dihydroxyphenyl) propanoic acid, 2-(2-acetyl-3,5-dihydroxyphenyl) acetic acid, 3-(4-hydroxy-3-methoxyphenyl) propanoic acid, Estriol, L-Threonine, and 5-Methyltetrahydrofolic acid (p < .05; Figure 5h). The significantly increased metabolites were mainly concentrated in prostaglandins and amines, and the significantly reduced metabolites were mainly concentrated in SCFAs, amino acids and estriol. Comparing the differences according to age and sex showed that the above metabolites have significant differences before and after eARGs addition in adult female and juvenile female groups (such as the differentially abundant metabolites phenethylamine, indole-3-acetic acid, 2-(2-acetyl-3,5-dihydroxyphenyl) acetic acid, 3-(4-hydroxy-3-methoxyphenyl) propanoic acid), but not in the adult male and juvenile male groups, indicating that eARGs have a marked impact on the metabolome of female mice.

### 2.6 Correspondence between metabolomics and cytokines reveals the affected pathways

**Figure 6.**
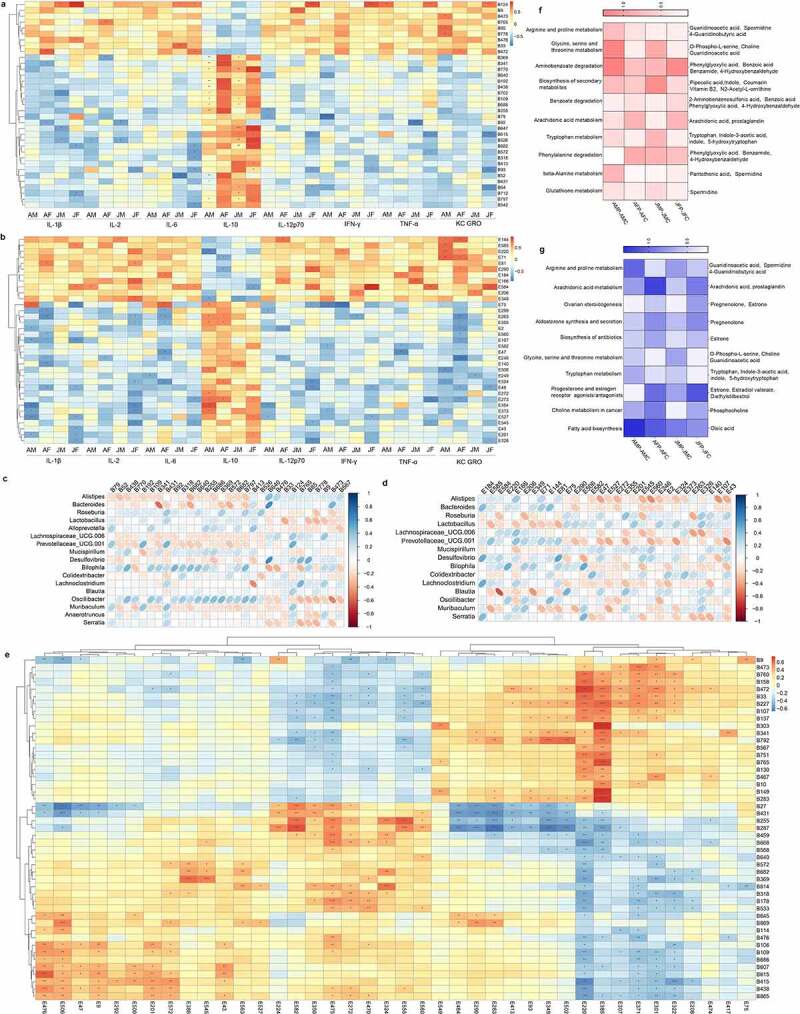
Analysis of the correlation between fecal and serum metabolites and other indexes. Heat map of the correlation analysis between fecal metabolites and serum immune cytokines of mice in the different age and sex groups (a). Heat map of the correlation analysis between serum metabolites and serum immune cytokines of mice in the different age and sex groups (b). Heat map of the correlation analysis between fecal metabolites and different intestinal microbiota of mice in the different age and sex groups. Blue indicates a positive correlation, red indicates a negative correlation, and blank indicates no statistical significance (p > .05) (c). Heat map of the correlation analysis between serum metabolites and intestinal microbiota of mice in the different age and sex groups (d). Heat map of the correlation analysis between fecal metabolites and serum metabolites (e). Heat map of pathways related to fecal differential metabolite enrichment of mice in different age and sex groups (f). Heat map of pathways related to serum differential metabolite enrichment in mice of the different age and sex groups (g). See Supplementary table 4 for the name of the metabolite corresponding to the metabolite ID in the figure.

The correlation analysis between serum immune cytokines and fecal differentially abundant metabolites showed that the metabolites that correlated positively with inflammatory cytokines were Arachidonic acid, Phenethylamine, 3-Methoxy prostaglandin F1α, Isoferulic acid, Prostaglandin E2, Sinapyl aldehyde, Paracetamol, 1-(4-hydroxyphenyl) propane-1,2-diol, Piperine, and N-Acetylserotonin, which mainly included aldehydes, alcohols and bases. The negatively correlated metabolites were 2-[2-oxo-2-(pyridin-3-ylamino) ethoxy] acetic acid, (2 R)-2-[(2 R,5S)-5-[(2S)-2-hydroxybutyl] oxolan-2-yl] propanoic acid, Ureidoisobutyric Acid, 3-Amino-4-methylpentanoic acid, Indole-3-acetic acid, 4’-Methoxyacetophenone, 2-piperidinobenzoic acid, 3-Indoleacrylic acid, 4’-Methoxyacetophenone, 4-Methyl-2-pentanone, N-α-L-Acetyl-arginine, and N-acetyl-L-ornithine, which are mainly classified as SCFAs, ketones and amino acids (Figure 6a).

The correlation analysis between serum immune cytokines and serum differentially abundant metabolites showed that the metabolites that correlated positively with inflammatory cytokines were Prostaglandin B2, Thromboxane aldehyde, Prostaglandin B1, 3-(2-thienyl) cinnoline-4-carboxylic acid, Stearamide, Phosphatidylcholine, Phosphatidylethanolamine, Prostaglandin A3, and Valine, which mainly included prostaglandins and amines. The negatively related metabolites were 3-(3,4-dihydroxyphenyl) propanoic acid, 3-(3,4,5-trimethoxyphenyl) propanoic acid, 2-(2-acetyl-3,5-dihydroxyphenyl) acetic acid, 4-(2,3-dihydro-1,4-benzodioxin-6-yl) butanoic acid, 3-(4-hydroxy-3-methoxyphenyl) propanoic acid, L-Threonine, gamma-Glutamyltyrosine, 4-Hydroxyisoleucine, 1-Methylhistidine, 5-Methyltetrahydrofolic acid, and Estriol, which are mainly classified as SCFAs, amino acids, and estrogen (Figure 6b).

The correlation analysis between the different microbiota and fecal differentially abundant metabolites showed that *Alistipes, Colidextribacter, Lachnoclostridium, Desulfovibrio* and other pathogenic bacteria correlated positively with Arachidonic acid, Phenethylamine, 3-Methoxy prostaglandin F1α, Isoferulic acid, Prostaglandin E2, Sinapyl aldehyde, Paracetamol, 1-(4-hydroxyphenyl) propane-1,2-diol, Piperine, and N-Acetylserotonin. Beneficial bacteria such as *Roseburia, Lactobacillus, Mucispirillum*, and *Blautia* correlated positively with 2-[2-oxo-2-(pyridin-3-ylamino) ethoxy] acetic acid, (2 R)-2-[(2 R,5S)-5-[(2S)-2-hydroxybutyl] oxolan-2-yl] propanoic acid, Ureidoisobutyric Acid, 3-Amino-4-methylpentanoic acid, Indole-3-acetic acid, 4’-Methoxyacetophenone, 2-piperidinobenzoic acid, 3-Indoleacrylic acid, 4’-Methoxyacetophenone, 4-Methyl-2-pentanone, N-α-L-Acetyl-arginine, and N-acetyl-L-ornithine (Figure 6c). Correlation analysis between the different microbiota and the differentially abundant metabolites in serum showed that harmful bacteria, such as *Alistipes, Colidextribacter, Lachnoclostridium* and *Desulfovibrio* correlated positively with metabolites including Prostaglandin B2, Thromboxane aldehyde, Prostaglandin B1, 3-(2-thienyl) cinnoline-4-carboxylic acid, Stearamide, Phosphatidylcholine, Phosphatidylethanolamine, Prostaglandin A3, and Valine. Beneficial bacteria, such as *Roseburia, Lactobacillus, Mucispirillum*, and *Blautia* correlated positively with 3-(3,4-dihydroxyphenyl) propanoic acid, 3-(3,4,5-trimethoxyphenyl) propanoic acid, 2-(2-acetyl-3,5-dihydroxyphenyl) acetic acid, 4-(2,3-dihydro-1,4-benzodioxin-6-yl) butanoic acid, 3-(4-hydroxy-3-methoxyphenyl) propanoic acid, L-Threonine, gamma-Glutamyltyrosine, 4-Hydroxyisoleucine, 1-Methylhistidine, 5-Methyltetrahydrofolic acid, and Estriol (Figure 6d). The above results showed that the differentially abundant and distributed metabolites of mice in different age and sex groups were reshaped after eARGs delivery to the intestine, while some metabolites are closely related to the inflammation-related gut microbiota.

Correlation analysis between fecal differentially abundant metabolites and serum differentially abundant metabolites showed that most of these metabolites in feces and serum demonstrated a significant positive correlation, indicating that the two metabolites might come from the same metabolic pathway, some of which are absorbed into the blood circulation and supplied to the body through intestinal epithelial cells (Figure 6e). The differential metabolites in feces were enriched in relevant signal transduction and metabolic pathways and signal transduction pathways according KEGG analysis. The results showed that they were enriched in the following metabolic pathways: Glycerophospholipid metabolism, Phenylalanine metabolism, Arginine and proline metabolism, Glycine, serine and threonine metabolism, Aminobenzoate degradation, Biosynthesis of secondary metabolites, Benzoate degradation, Lysine degradation, Phenylalanine metabolism, Aminobenzoate degradation, Tryptophan metabolism, Arginine and proline metabolism, Glutathione metabolism, and beta-Alanine metabolism. Among them, metabolites in adult females and juvenile females were enriched more in the arachidonic acid metabolic pathway, and fewer metabolites were enriched in the tryptophan metabolic pathway (Figure 6f). KEGG analysis of the differentially abundant metabolites in serum showed that they were enriched in the following metabolic pathways: Biosynthesis of polyketide, Cortisol synthesis and secretion, Cushing’s syndrome, Citrate cycle (TCA cycle), Biosynthesis of antibiotics, Aldosterone synthesis and secretion, Biosynthesis of unsaturated fatty acids, Microbial metabolism in diverse environments, Ovarian steroidogenesis, Androgen and estrogen receptor agonists/antagonists, Choline metabolism in cancer, Glycerophospholipid metabolism, Microbial metabolism in diverse environments, Fatty acid biosynthesis. Among them, in adult females and juvenile females, fewer metabolites were enriched in Glycine, serine and threonine metabolism, fatty acid metabolic pathway, while more metabolites were enriched in the estrogen synthesis related metabolic pathway. In adult females and juvenile females, fewer metabolites were enriched in the Arachidonic acid metabolism and Progesterone and estrogen receptor agonists/antagonists pathways (Figure 6g). The above results showed that eARGs had a significant impact on arachidonic acid metabolism and tryptophan metabolism in adult female and juvenile female groups, which are associated with inflammatory cytokine responses.

### 2.7 Prediction of intestinal damage in mice

**Figure 7.**
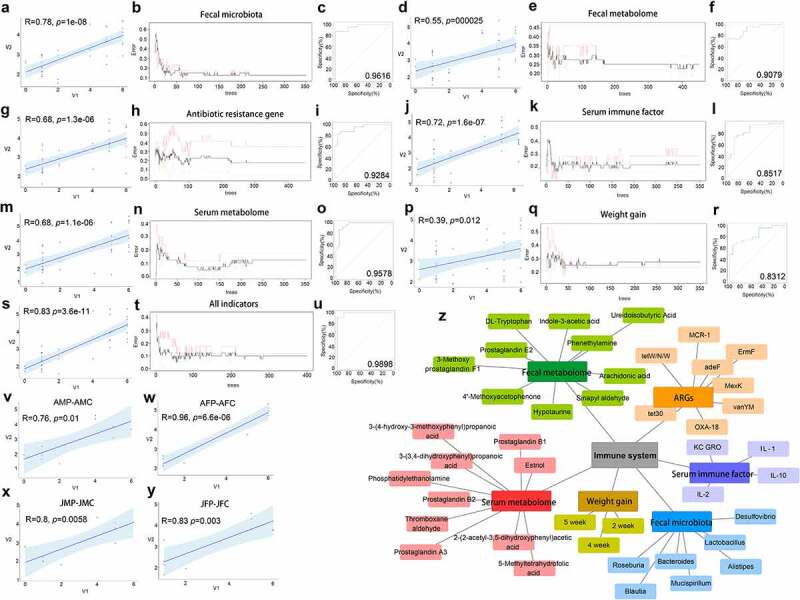
The effect and contribution of different detection indexes to predict the immune state of mice. Scatter plot constructed using Spearman correlation analysis, the correlation between different detection indexes and immune status is stronger when R is closer to 1, and the smaller the p value, the stronger the significance (a, d, g, j, m, p). The influence of the number of decision trees on the error rate. The x-axis represents the number of decision trees, and the y-axis represents the error rate. When the number of decision trees is approximately 400, the error rate is relatively stable (b, d, h, k, t).Verification of the receiver operating characteristic (ROC) curve results using the five-time cross-validation model. The area under the ROC curve (AUC) is close to 1, indicating that the model has strong stability (c, f, i, l, o, r, u). Scatter plot by Spearman correlation analysis between different detection indexes and the immune status of mice of same age and sex (v, w, x, y). Network diagram between the factors that have a greater contribution to the immune state and the immune system of the body in different detection indexes (z) .

Using the random forest model algorithm, the immune state (the degree of intestinal pathological injury) of mice was predicted through six detectable indexes of mice: intestinal microbiota, fecal metabolome, intestinal resistance genes, serum immune factor, serum metabolome, and body weight, to establish a prediction model for each index. We found that the order of prediction efficiency of each index was intestinal microbiota > serum metabolic group > intestinal resistance genes > fecal metabolic group > serum immune factor > weight change, and the prediction effect using all six indexes was the best (p = 3.6e-11; error = 0.1; area under the ROC curve (AUC) = 0.9898; Figure 7a-u). The above results showed that the more detection indexes, the better the prediction effect. If a single index was selected for prediction, the intestinal microbiota, serum immune cytokine, serum metabolism group, and gut antibiotic resistome had a better prediction effect than the other indexes. We also used these six indicators for prediction in the different age and sex groups. We found that all age and sex groups are applicable to the above prediction model, adult male (p = .01; Figure 7v), adult female (p = 6.6e-06; Figure 7w), juvenile male (p = .0058; Figure 7x), juvenile female (P = .003; Figure 7y). Among them, adult females had the best prediction effect, followed by juvenile females, and finally juvenile and adult males.

According to the comprehensive analysis, all six indicators have bacteria or molecules that contribute greatly to the prediction of immune system damage: Fecal microbiota such as *Alistipes, Bacteroides, Roseburia, Lactobacillus, Mucispirillum, Desulfovibrio, Blautia*; fecal metabolites including Arachidonic acid, Phenethylamine, 3-Methoxy prostaglandin F1α, Sinapyl aldehyde, Prostaglandin E2, Indole-3-acetic acid, DL-Tryptophan, 4’-Methoxyacetophenone, Ureidoisobutyric Acid, and Hypotaurine; Antibiotic resistance genes including *adeF, ErmF, tetX, aadA6/aadA10, sdiA, vanUG, oleC*, and *APH3-Ia*; serum immune cytokines including IL-2, IL-1β, IL-10, and KC/GRO; serum metabolites including Prostaglandin B2, Thromboxane aldehyde, Prostaglandin A3, Phosphatidylethanolamine, Prostaglandin B1, 3-(3,4-dihydroxyphenyl) propanoic acid, 2-(2-acetyl-3,5-dihydroxyphenyl) acetic acid, 3-(4-hydroxy-3-methoxyphenyl) propanoic acid, Estriol, and 5-Methyltetrahydrofolic acid; and weight gain including 2, 4, and 5 weeks (Figure 7z). The above indicators are also markers with significant differences from the previous analysis, indicating that with the growth of the body, the changes in the microbiota, metabolic function, and the resistome will have an important function in the induction of inflammation and intestinal injury in mice.

### 2.8 Ex vivo *and* in vivo *immunomodulatory effects of microbial metabolites*

**Figure 8.**
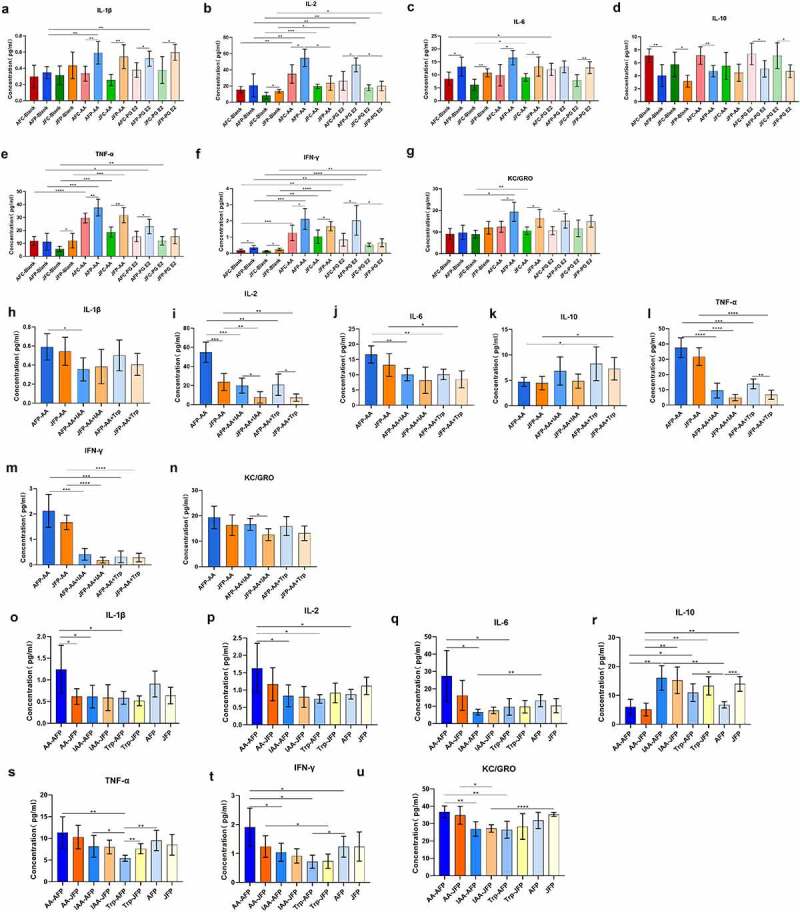
Effects of different metabolites on peripheral blood mononuclear cells (PBMCs) and the intestinal structure of the mice. Histogram of cytokine concentrations in the supernatant of cell culture when arachidonic acid and prostaglandin E2 were added to PBMCs from the adult female treated with plasmid group and the control group, and juvenile female treated with plasmid group and the control group (a-g). Histogram of cytokine concentrations in the supernatant of cell culture when indole-3-acetic acid and tryptophan were added to the PBMCs of adult female and juvenile female mice on the basis of arachidonic acid stimulation (h-n). Histogram of cytokine concentrations in the serum of adult female and juvenile female with inflammation fed arachidonic acid, indole-3-acetic acid, and tryptophan, respectively, for one week (o-u).

Our results showed that the eARGs had the most significant effect on adult female and juvenile females; therefore, female mice were selected to verify the regulatory effect of intestinal microbiota metabolites on immune function. We selected arachidonic acid (AA) and prostaglandin E2 (PGE2) in the arachidonic acid metabolic pathway and indole-3-acetic acid (IAA) and tryptophan (Trp) in the tryptophan metabolic pathway. The results of *in vitro* experiments showed that when no metabolites were added, the inflammatory factors secreted by peripheral blood mononuclear cells (PBMCs) in the adult female mice treated with the plasmid group (AFP) and the juvenile females treated with the plasmid group (CFP) were higher than those in their respective control groups (AFC and CFC). Among them, IL-6 and IFN-γ levels in the AFP group increased significantly, while IL-10 levels decreased significantly (p < .05). The IL-2, IL-6, TNF-α, and IFN-γ levels in the CFP group increased significantly, while IL-10 levels decreased significantly (p < .05), indicating that eARGs can reshape the mouse immune phenotype (profile) after entering the intestine, which shows that there is a difference in the secretion of immune cytokines. When AA and PGE2 were added to stimulate PBMCs, compared with those on the control group, the levels of IL-1β, IL-2, IL-6, TNF-α, IFN-γ, and KC/GRO increased in the AFP group significantly, and IL-10 levels decreased significantly (p < .05) after AA stimulation. The levels of IL-1β, IL-6, TNF- α, IFN- γ, and KC/GRO increased significantly in CFP group when stimulated by AA (p < .05), and the IL-10 level decreased significantly (p < .05). Compared with those in the CFC group, the levels of IL-1β, IL-2, TNF-α, IFN-γ, and KC/GRO showed significant increases in the AFP group when stimulated by PGE2, while the IL-10 level decreased significantly (p < .05). The levels of IL-1β, IL-6, IFN-γ and KC/GRO showed significant increases in the CFP group when stimulated by PGE2, while the IL-10 level decreased significantly (p < .05, Figure 8a-g). The above results showed that AA and PGE2 could significantly promote the secretion of pro-inflammatory cytokines by single cells, reduce the secretion of anti-inflammatory cytokines, and had a more prominent regulatory effect on the eARGs-treated group, indicating that the immune cells of mice treated with eARGs will be more sensitive to external stimuli, which is also consistent with our previous conclusions. Taken together, the variation in cytokine production suggested that the eARGs might affect mouse immune responses.

On the basis of the above results, we selected the AA stimulation group, with a more significant effect, for recovery by the addition of indole-3-acetic acid and tryptophan, respectively. The results showed that the levels of IL-1β, IL-2, IL-6, and TNF- α in the AFP group added with indole-3-acetic acid were significantly lower compared with those in the AFP group stimulated by AA. IFN-γ, IL-2, and TNF-α levels in the CFP group added with indole-3-acetic acid decreased significantly compared with those in the CFP group stimulated by AA. IL-2, IL-6, TNF-α, and IFN-γ levels in the AFP and CFP groups added with tryptophan were significantly lower than those in the AFP and CFP groups stimulated by AA. In addition, IL-10 levels in the AFP and CFP groups added with tryptophan were significantly higher than those in the AFP and CFP groups stimulated by AA (p < .05, Figure 8h-n). The above results showed that both indole-3-acetic acid and tryptophan could significantly improve the activity of immune cells stimulated by arachidonic acid, which is manifested by increased production of anti-inflammatory cytokines and reduced secretion of pro-inflammatory cytokines.

After the *in vitro* cell experiments, we selected arachidonic acid, indole-3-acetic acid, and tryptophan for *in vivo* experiments, and gavaged these three metabolites into mice with inflammation caused by eARGs. Compared with the blank recovery group, IL-2 and IFN- γ levels in the AFP group after intragastric administration of AA increased significantly (p < .05), and the IL-10 level in the CFP group after intragastric administration of AA decreased significantly (p < .05). IL-2 and IFN- γ levels in the AFP group after intragastric administration of indole-3-acetic acid decreased significantly compared with those in the blank recovery group, and IL-1β, IL-2, IL-6, TNF- α, IFN- γ, and KC/GRO levels decreased significantly compared with those in the AFP group fed AA, while IL-10 was significantly higher than that of the AFP group fed AA (p < .05). After intragastric administration of indole-3-acetic acid, the KC/GRO level of the CFP group decreased significantly compared with that in the blank recovery group, and the IL-10 level increased significantly compared with that of the CFP group after intragastric administration of AA (p < .05). TNF- α and IFN- γ levels in the AFP group after intragastric administration of tryptophan were significantly lower than those in the blank recovery group, while the IL-10 levels was significantly higher than that in the blank recovery group. After feeding tryptophan, the IL-1β, IL-2, IL-6, IFN-γ, and KC/GRO levels decreased significantly compared with those of the AFP group fed AA, while IL-10 was significantly higher than that of the AFP group fed AA (p < .05). After intragastric administration of tryptophan, the IFN- γ in levels in the CFP group decreased significantly compared with that of the CFP group fed AA, while the IL-10 level increased significantly compared with that of the CFP group fed AA (p < .05, Figure 8o-u). These results showed that tryptophan and indole-3-acetic acid could improve the immune function damage caused by eARGs. This was consistent with the cell experiment, i.e., arachidonic acid can further aggravate intestinal inflammation in mice, while tryptophan and indole-3-acetic acid can alleviate this inflammation. Thus, metabolites regulated by the intestinal microbiota driven by eARGs contribute to variability in immune responses.

We next analyzed each intestinal segments of the mice and found that the structure of each intestinal segment of mice gavaged with AA was altered. We observed a slight abnormality in the overall structure of small intestine and in the mucosal layer, and a small amount of inflammatory cell infiltration in villi and crypts. The overall structure of the cecum tissue was abnormal, accompanied by the presence of goblet cells, and many inflammatory cells in the mucosal layer and submucosa. There was slight abnormality in the colon tissue, the mucosal layer, goblet cells, ectopic crypt glands, and many infiltrated inflammatory cells in the submucosa. After intragastric administration of indole-3-acetic acid and tryptophan, all the intestinal segments of mice showed restoration to a certain extent. The small intestine tissue and the mucosal layer were basically normal, goblet cells were visible, and a few infiltrated inflammatory cells were visible in the villi and crypts, with no abnormality in the submucosa and muscular layer. Furthermore, there was a slight abnormality in the cecum tissue and the mucosal layer, goblet cells were observed, and a few infiltrated inflammatory cells were visible in the mucosal layer, with no abnormality in the submucosa and muscular layer. The colon tissue was basically normal; however, the mucosal layer was slightly abnormal. Hyperemia points could be seen in some villi, together with goblet cells and a few infiltrated inflammatory cells. In the recovery group without external factor intervention, all intestinal segments of mice were also partially recovered. The small intestinal tissue and the mucosal layer were slightly abnormal, individual villi were denatured, the number of goblet cells was increased, a few infiltrated inflammatory cells were seen in the villi and crypts. The cecum tissue and mucosal layer were slightly abnormal, focal mucosal ulceration and goblet cells could be observed, a few infiltrated inflammatory cells were seen in the mucosal layer. The colon tissue and the mucosal layer were slightly abnormal, goblet cells could be seen, along with ectopic crypt glands and inflammatory cell infiltration in the submucosa (**Fig. S1b, Table S1**). The above results showed that continuous stimulation by AA further aggravated the inflammation caused by eARGs, and indole-3-acetic acid and tryptophan could aid intestinal tissue repair and reduce inflammation. When the external stimulation was relieved, the recovery group with no intervention could recover to a certain extent; however, the effect was less than that in the indole-3-acetic acid and tryptophan assisted recovery groups.

## 3. Discussion

The World Health Organization (WHO) declared that antimicrobial resistance (AMR) is a global public health threat, and a leading cause of death worldwide.^17^ Therefore, high-risk ARGs that pose significant threats to human health were identified using a new strategy,^18^ in which the *mcr-1* gene was assigned to Rank I (the highest risk). Previous studies largely focused on the abundant ARGs, the transmission of ARGs between the environment, humans, and animals,^19^ as well as investigating their association with human disease and health indirectly in human cohorts. Shuai *et al*. uncovered the link between gut microbial ARGs and progression of type 2 diabetes in a large human cohort,^20^ while cirrhosis is associated with a high gut microbial ARGs burden, which worsens with disease progression.^21^ However, there is limited knowledge on how eARGs shape the gut microbiome and cytokine responses after entry into the intestine. Therefore, identifying the interplay between eARGs, microbes, metabolites, and the gut antibiotic resistome associated with immune system regulation is of great importance to tackle this global problem, providing directions to therapeutic strategies or dietary interventions. Herein, we investigated the impact of a plasmid harboring colistin resistant eARG *mcr-1* of mice in different age and sex groups.

Mice of different age and sex have unique microbiota markers, and the entry of eARGs into the intestine will have a significant impact on the mouse’s original gut microbiota. Following entry of eARGs into the intestine, they will interact with a wide variety and abundant gut microbiota. The gut microbiota has an individualized bacterial structure in each mouse, and eARGs can affect or even remodeled it. Specific to the different microbiota, the results showed that *Alistipes* had high abundance in each group, and they increased significantly after the addition of eARGs. Studies have showed that *Alistipes* might promote the occurrence of certain diseases, such as colitis, is pathogenic in colorectal cancer, and is related to depression.^22^ Sulfate reducing bacteria (SRB) are anaerobic bacteria that can reduce sulfate and produce H_2_S. Among them, *Desulfovibrio* (DSV) is the dominant SRB in the human colon. Endogenous H_2_S will poison intestinal epithelial cells; therefore, researchers have speculated that there is a certain relationship between DSV and intestinal diseases, indicating that an increase in intestinal DSV is an important feature of polyps and ulcerative colitis.^23^ Following eARG entry into the intestine, these bacteria were increased significantly, which might be crucial to promote intestinal inflammation.

The numbers of certain intestinal probiotics decreased significantly in each experimental group, including *Roseburia, Eubacterium, Butyricicoccus*, and *Oscillibacter. Roseburia* is a common butyric acid producing bacterium and one of the key bacteria that degrade dietary fiber xylan in human intestine.^24^ Some members of *Eubacterium* produce butyrate, which plays a key role in energy balance, colon movement, immune regulation, and inhibition of intestinal inflammation. *Eubacterium* converts bile acids and cholesterol in the intestine, thereby promoting its homeostasis.^25^ *Butyricicoccus* ferments sugars to form a lse amount of butyric acid that can reduce the permeability of intestinal epithelium and enhance the proliferation of intestinal epithelial cells by raising the expression of tight junction proteins, thereby lengthening the villi and enhancing their absorption capacity.^26^ The low calorie Mediterranean diet (MD) can significantly change the abundance of *Enterorhabdus, Lactobacillus*, and *Bacillus p-hydroxybenzoate*, and the change of SCFAs is negatively correlated with the production of some inflammatory cytokines (such as VEGF, MCP-1, IL-17, IP-10, and IL-12) following MD consumption. Changes in the production of SCFAs support their function in regulating the inflammatory cytokines response, thereby mediating the protective and anti-inflammatory effects of the MD.^27^ *Oscillibacter* belongs to *Firmicutes*, and some studies have shown that the abundance of *Oscillibacter* in the intestines of healthy people is significantly higher than that of people with Crohn’s disease, and the main metabolic end product of *Oscillibacter* is valeric acid,^28^ suggesting that *Oscillibacter* has potential anti-inflammatory effects.^29^ However, the production of SCFAs by these bacteria was decreased significantly, making the intestine lose its protective function, resulting in inflammation.

In addition, some other differentially abundant bacteria play a vital role in diseases other inflammation. For example, the abundance of the *Prevotella* is higher in males than in females, which is a biological phenomenon across multiple species and populations. In a population study, *Prevotella* was determined to be more abundant in men than in women, based on 16S rRNA targeted oligonucleotide probes.^30^ Another study showed that men were three times more likely to have the characteristic intestinal type than women, consisting of fewer *Bacteroides* and higher levels of *Prevotella*,^31^ of which the main fermentation products are acetic acid and succinic acid, together with small amounts of isobutyric acid, isovaleric acid and lactic acid. These metabolites are conducive to protecting the intestine, which might partially explain why in our study, the degree of intestinal inflammation in males was lower than that in females. After eARG treatment, the body weight of some mice decreased significantly, and the results showed that some bacteria decreased significantly, such as *Dorea, Blautia*, and *Ruminococcus*. The two major hormones of adipocytes are leptin and adiponectin. A study owed that *Dorea* correlates positively with the concentration of leptin in the body and negatively with the concentration of adiponectin. In that study, significantly higher *Dorea* levels were observed in the obese group compared with those in the control group.^32^ *Blautia, Ruminococcus, Clostridium* levels also correlated positively with body weight.^33^ In addition, *Parabacteroides* is one of the core bacteria in the human body. Correlation analysis showed that its content correlated negatively with obesity,^34^ nonalcoholic fatty liver disease, and diabetes, suggesting that it might play a positive role in regulating glucose and lipid metabolism.

In many environments, stochastic processes usually play a key role in shaping the microbial community structure. After ARGs entry into the intestine, our results showed that the assembly of the gut microbial community was significantly impacted; however, the stochastic processes still had an essential role in shaping the microbiota in mice. The results showed that the influence of eARGs varied in mice of different ages and sexes, and it had a greater impact on the female group. The value of NCM parameter R^2^ was significantly lower in the experimental mice, and the R^2^ decreased most significant in the female groups, while the bacterial dispersal in experimental mice is likely to be higher than control groups according to the calculated Nm values. In addition, regarding the community migration rate, the m values in the experimental groups were higher than those in the control groups, indicating the increased dispersal ability of most taxa in the experimental groups. These differences might be attributed to eARGs, which significant remodeled the structure of the gut microbial communities and elevated their dispersal ability, making the intestinal microenvironment conducive to the diffusion of the eARGs, which further increase their detrimental effects on host health. Although the changes in the gut microbial communities were strongly driven by stochastic processes, the taxa that were above and below the neutral prediction might also be structured by deterministic processes, another complementary mechanism.^35^ Further microbial community ecology research might help to fully understand the mechanisms of microbial community assembly following eARG entry into the mouse intestine.

In addition to the alteration of the composition and assembly of the gut microbial community resulted from eARGs treatment, the gut antibiotic resistome also changed significantly. The results showed that after eARGs treatment, the abundance of ARGs biomarkers in the gut microbiome increased significantly. Interestingly, we observed that *mcr-1* correlated positively with inflammatory cytokine levels (Figure 4j), indicating that it had invaded into gut microbial community. Moreover, the significant shifts in the gut microbial composition after *mcr-1* addition, and the increase in the abundance of genes enriched in plasmid transfer and diffusion pathways, formed an intestinal microenvironment that might promote the spread of ARGs.^36^ These ARGs biomarkers were distributed in different bacteria, especially probiotics, opportunistic pathogens, and intestinal symbionts, which might interact with the host and result in damaged health.

Alterations of the gut microbiota led to dysregulation of the fecal and serum metabolomes. The differentially abundant metabolites were mainly prostaglandins, estrogens, amines, aldehydes, alkaloids, amino acids, SCFAs, and ketones, some of which have significant correlations with inflammatory cytokines and the intestinal microbiota. Several metabolites have been shown to influence colitis.^37^ In our study, prostaglandins in the fecal and serum metabolomes increased significantly after eARGs treatment. It has been reported that the level and profile of prostaglandin production will change significantly during the inflammatory response. In tissues without inflammation, prostaglandin production is usually very low; however, in acute inflammation before leukocyte recruitment and immune cell infiltration, prostaglandin production increases immediately.^38^ In addition, carbohydrates are completely digested after entering the intestine, which will produce abundant metabolites, including stearic acid, arachidonic acid, and indole sulfuric acid. These proinflammatory metabolites signal the intestine to the change the levels of tight junction proteins, thereby increasing the permeability of the intestines and continuously stimulating the intestinal mucosa. This leads to an inappropriate immune response, ultimately causing intestinal inflammation.^39^

Further metabolite changes showed that the SCFAs in the fecal and serum metabolomes were significantly downregulated after eARGs treatment. SCFAs can bind to specific receptors on epithelial cells and myeloid cells (such as dendritic cells (DC)), to enhance their tolerance and promote the production of regulatory T cells (Treg) .^40^ In mice, the SCFA propionate promotes colonization resistance to *Salmonella* Typhimurium by disrupting bacterial pH homeostasis.^41^ Butyrate, another SCFA, can reduce the expression of typical pro-inflammatory mediators (NOS2, COX-2, IL-6), pro-inflammatory adipokines (lipocalin-2 and nesfatin-1), and adhesion molecules (VCAM-1 and ICAM-1), and inhibits a variety of inflammatory signaling pathways (NF-κB, MAPK, AMPK-α, PI3K/Akt) .^42^ In addition, butyrate can lead colonic cells to consume oxygen via a metabolic pathway that acts through β-oxidation. By maintaining the anaerobic environment in the colon of mice, butyric acid is conducive to the growth of beneficial *Clostridium* and obligate anaerobic bacteria (including butyric acid producing bacteria) and restricts the growth of pathogenic *Enterobacteriaceae*.^43^ Therefore, an imbalance of the intestinal microbiota might be the basis of many human diseases.^44^

In mice, the prebiotic microbiota-related metabolites spermine and histamine have been shown to inhibit the NLRP6 inflammasomes in intestinal epithelial cells and reduce the production of colonic inflammatory response.^45^ In addition, the intestinal microbiota directly affects a variety of chemical messengers through the synthesis of proteins, including neurotransmitters, such as serotonin and dopamine.^46^ In addition, tryptophan-derived metabolites, such as indole and kynurenine, and several bacteria, can convert tryptophan into indole and indole derivatives.^47^ As early as 1897, tryptophan was found to be converted to indole 70 through *Escherichia coli* and *Vibrio cholera*.^48^ Moreover, *Clostridium sporogenes* converts tryptophan to tryptamine, indoleacetic acid (ILA), and indole propionic acid (IPA) .^49^ In the intestine, tryptamine can induce intestinal chromaffin cells located on the mucosal surface to release the neurotransmitter serotonin (5-HT). 5-HT stimulates gastrointestinal motility by acting on neurons in the intestinal nervous system.^50^ Correlation analysis between the human intestinal microbiome and the *in vitro* cytokine response in whole blood after microbial stimulation indicated that there is a negative correlation between IFN-γ and the bacterial genes responsible for the conversion of tryptophan to indole ethanol (IE), indicating that IE has anti-inflammatory properties.^51^ A recent study found that indoleacrylic acid has anti-inflammatory and antioxidant effects on LPS-activated human PBMCs, expressed as decreased secretion of IL-6 and IL-1β and activation of the NRF2-ARE pathway,^52^ which is considered to be a therapeutic target for the prevention of neurodegenerative diseases^53^ and IBDs.^54^ Similarly, indoleacetic acid and tryptamine attenuated the pro-inflammatory cytokine response in mice. In addition, mouse macrophage culture and hepatocyte culture are highly dependent,^55^ suggesting that microbial tryptophan catabolism might also affect the inflammatory response of the liver. Over the last 10 years, IBD related to the intestinal microbiota has been a research focus. Interestingly, in patients with IBD, levels of serum tryptophan were markedly decreased compared with those in healthy controls, and in patients with Crohn’s disease (CD), the levels of serum tryptophan were markedly lower than those in patients with ulcerative colitis (UC).^56^ Our metabolic pathway analysis showed that female mice and pups were less enriched in tryptophan metabolism and fatty acid synthesis pathways.

Compared with the mice in control groups, the variation in cytokine production in the experimental mice clearly suggested that the eARGs regulated immune responses, possibly via metabolites. Among the eight cellular immune cytokines detected, the levels of seven pro-inflammatory factors increased to a certain extent after eARGs treatment, among which IL-1β was most significant. IL-1 mediates the expression of many secondary inflammation-related genes. IL-1 response genes coordinate local inflammation, including the attraction and activation of adaptive immune system cells at the infection site.^57^ In the present study, IL-12p70 increased significantly in the experimental groups. IL-12p70 could promote Th0 cells to differentiate into Th1 cells and promote IFN-γ production, and Th1 cytokines, such as IL-2, were synthesized, while Th2 cells were inhibited from synthesizing IL-4 and IL-10. Th1 cytokines (IFN-γ, IL-2) promote cellular immunity, particularly activating monocytes, natural killer cells (NKs), and cytotoxic T lymphocytes, while the increase in Th2 cytokines induces immune tolerance. The chemokine KC/GRO is composed of LPS and pro-inflammatory cytokines IL-1 and TNF, and through NF-κB signaling, is the main chemokine induced in ovarian surface epithelial cancer cells.^58^ Furthermore, the anti-inflammatory cytokine IL-10, which was significantly reduced after eARG treatment, plays a core role in infection by limiting the immune response to pathogens, thereby preventing damage to the host.^59^ In addition, the age and sex characteristics of the host also have an important impact on inflammation.^51^ Our results clearly showed that the eARGs had a more significant influence on the cytokine responses of female mice after entry into the intestines, which was consistent with the alteration of the microbiota and metabolomes.

The immune cells in the blood can infiltrate the intestinal mucosa and submucosa, contributing to intestinal inflammation.^60^ Moreover, when the intestinal tract is stimulated by detrimental microbial metabolites, it will also damage peripheral blood immune cells. PBMCs with characteristic immunological profiles, formed after eARG treatment, were cultured with different metabolites to verify the response of these immune cells to stimulation. We observed that prostaglandin E2 and arachidonic acid were significantly upregulated in eARGs-induced inflammation, and had the characteristics of initiating an inflammatory response during co-culture with immune cells. The occurrence of inflammation is usually self-limiting; however, when the stimulation persists, the regression mechanism of inflammation may fail, resulting in excessive or persistent inflammation.^61^ Metabolic stress leads to inflammation, and inflammation itself destroys the metabolic balance. Therefore, chronic metabolic or inflammatory diseases usually show a vicious feedforward cycle of metabolic disorders and inflammatory reactions.^62^ Endogenous arachidonic acid is mainly released through cell membrane phospholipids, which is catalyzed by enzymes of the phospholipase A2 superfamily and is induced by various cell activation signals, including stimulation of the tumor necrosis factor receptor and Toll like receptor 4, driven by inflammation or infection.^63^ In addition, in the AA metabolic pathway, cyclooxygenase 1 (COX1) and cyclooxygenase 2 (COX2), also known as prostaglandin G/H synthase, promote the production of thromboxane A2 (TXA2), prostacyclin (PGI2), and several prostaglandins (PG).^64^ COX1 is expressed in all tissues susceptible to lipopolysaccharide-induced inflammation.^65^ Its main metabolites are prostaglandin E2 (PGE2), PGI2, PGD2, and PGF2α, which can regulate the host immune response, vascular tone, thrombosis and other physiological processes. Therefore, prostaglandins are one of the most effective pro-inflammatory mediators.^66^ Tryptophan can regulate the intestinal inflammatory response, mainly by promoting protein turnover, repairing damaged epithelial cells, and producing antibacterial peptides to alleviate the intestinal inflammatory response.^67^ The catabolic pathway of tryptophan is the key regulatory mechanism to maintain intestinal immune tolerance. Its promotion of intestinal immune tolerance is mainly reflected in two aspects: First, macrophages and DCs cause tryptophan deficiency in T cells by increasing tryptophan consumption, thereby inhibiting T cell proliferation. Second, tryptophan metabolites might inhibit the activity of specific immune cells by promoting apoptosis.^68^ In the metabolic pathway of tryptophan, tryptophan produces indole-3-acetic acid through deamination and decarboxylation, and tryptophan is degraded into nicotinic acid, pyruvate, and acetyl coenzyme a through the formation of canine urinary tryptophan.^69^ Studies have shown that indole-3-acetic acid inhibits the migration of bone marrow-derived macrophages to monocyte chemoattractant protein-1 in a dose-dependent manner. Indole-3-acetic acid not only has the ability to reduce macrophage inflammation, but also significantly inhibits genes expression and the secretion of pro-inflammatory cytokines;^55^ therefore, the tryptophan-derived bacterial metabolite indole-3 acetic acid can also be used to reduce epithelial cell inflammation.

In addition to their impact on the overall immune system, the eARGs also contributed to the metamorphosis of the structure of each intestinal segment of mice in the different age and sex groups. In all the mice in each group, the most significant alterations in the overall structure occurred in the cecum, indicating that the cecum might be the main site for further expansion and transfer of exogenous particles in the intestine. In addition, some inflammatory cells were observed in the mucosal layer of the cecum. This inflammatory environment provides favorable conditions for the expansion and transfer of eARGs by changing the permeability of bacterial membrane, inducing the SOS response, and changing the composition and diversity of bacteria, which is also in line with our previous research.^36^ We observed slight abnormalities in the overall structure of the colon and small intestines of the mice, accompanied by fewer inflammatory cells accumulated in the mucosal layer.

The eARGs had a greater impact on the immune system of female mice, which is consistent with the conclusions of Horst et al.^70^ who stated that adult female immunity is more susceptible to autoimmune diseases resulting from external stimulations. In addition, special stressors during prolonged puberty in female mice lead to lasting changes in the brain’s behavioral response to estradiol and progesterone. Specific adverse experiences in females might lead to long-term changes in the brain’s response to estradiol and/or progesterone by activating the immune system.^71^ Therefore, under the action of estrogen, the immune system of female mice might be more vulnerable to external factors, which is consistent with our results that the effect of the eARGs on the immune system was more significant in adult females.

In this study, we used a machine learning method to predict the intestinal injury after ARGs treatment using information for the intestinal microbiota, the serum metabolome, intestinal resistance genes, the fecal metabolome, serum immune factors, and weight change. The results showed that the prediction accuracy based on the intestinal microbiota was the highest, up to 96.16%, which further confirmed that eARGs lead to immune dysfunction and ultimately damage the body by changing the intestinal microbiota and metabolic function. The intestinal microbiota has been widely used to predict host characteristics and status. For example, Salosensaari found that the intestinal microbiota could predict the risk of human etiological specific death.^72^ Loomba *et al*. used the intestinal microbiota to predict advanced fibrosis in human nonalcoholic fatty liver.^73^ Zhu et al. used the intestinal microbiota to track the source of *Pseudosciaena crocea* (wild or cultured).^35^ In the present study, we predicted the microbiota assembly mechanism (via a deterministic or stochastic processes) using a species community neutral model. We found that the randomness of the community disturbed by environmental factors decreased and the species mobility increased in the assembly process, allowing a comparison before and after the impact of eARGs. Therefore, the intestinal microbiota can be used as an accurate and noninvasive index to predict the intestinal injury caused by eARGs entering the body. At the same time, those members of the microbiota with a large contribution provide feasible targets to prevent and control the health damage caused by eARGs.

AMR is a “silent epidemic”, which is more lethal than malaria and AIDS. The results of this study showed that eARGs will significantly change the original intestinal microbiota structure, abundance, and community assembly after delivery into the mouse intestines. The changes in the microbiota will be followed by changes in microbiota-mediated metabolic pathways, resulting in the production of different metabolites. The most significant changes are the upregulation of pro-inflammatory prostaglandins and the downregulation of anti-inflammatory tryptophan and SCFAs. These metabolites might have contributed to the damage to the structure of the intestinal mucosal barrier. The serum pro-inflammatory cytokines were significantly modulated, leading to systemic and local intestinal inflammation, and had a certain influence on the growth of the mice. In addition, the impact of the eARGs varied in the mice in the different age and sex groups, the results showed that the immune system of adult females is the most vulnerable to the eARGs, followed by juvenile females. As far as we know, this was the first study to investigate how eARGs affect host health via the gut microbiota, and provides new perspectives for optimizing strategies to tackle this global problem. However, the limitation of this study was that there are obvious differences between the mouse and human gut microbiota. How to transfer the experimental results of mouse models to humans remains a problem that needs careful consideration in current research on the relationship between the intestinal microbiota and human diseases. There are differences in intestinal structure between mouse and human, especially with regard to the stronger fermentation ability of mouse cecum, which might affect the diversity and composition of the intestinal microbiota.^74^ Studies have shown that few bacterial species are shared between the gastrointestinal tracts of humans and mice.^75^ Through the whole genome approach, the mouse gastrointestinal bacteria catalog (MGBC) was compared with the human gastrointestinal genome (UHGG),^76^ which revealed that only 2.58% (103/3997) of species are shared between the human and mouse gut microbiotas. Of these shared species, 93.2% could be assigned a species rank by the Genome Taxonomy Database Toolkit, and 55.3% have cultured representatives within the MGBC.^77^ Although few species are shared, functions at the gene level show that up to 95% of intestinal microbial functions are shared between humans and mice.^78^ The intestinal ecosystems of the two hosts shared 84.5% of the KEGG orthology groups and 82.1% of the InterPro protein families.^77^ Therefore, mice can partly simulate the function of the human microbiota. However, to further translate the research results into the treatment of human diseases, we also plan to transplant a human microbiota into the intestines of sterile mice for research and to collect human microbiota samples for a cohort study to further reveal the impact of eARGs on humans.

## 4. Materials and methods

### 4.1 Animal experiments

In this experiment, 7-week-old adult Balb/c male and female mice and young (3 weeks old) Balb/c female and male mice (Beijing Weitonglihua Experimental Animal Company, Beijing, China) were raised in specific pathogen free (SPF) barrier facilities. The mice had free access to food and water and were weighed every week. We divided the four groups of mice into control groups and experimental groups. The *mcr-1* plasmid was extracted from *E. coli* J53 Azi^R^ conjugants and used for experiments (Supplementary Materials and methods). In the experimental group, each mouse was gavaged with 200 μL of *mcr-1* plasmid solution (10^9^ copies) once a day, while the control group was gavaged with 200 μL of normal saline once a day. Gavage was carried out continuously for 4 weeks. Subsequently, some of the mice in the experimental groups were gavaged with arachidonic acid, tryptophan, or indole-3-acetic acid for 2 weeks, respectively. During this period, the fresh feces of the mice were collected within four hours into a high-pressure sterilized centrifuge tube, and immediately stored at – 80°C. At the end of the experiment, mouse serum was collected and stored at – 80°C. The mice were then sacrificed humanely and their intestines were dissected out for further experiments. All the experimental procedures complied with the ARRIVE guidelines and were approved by the Ethical Committee for the welfare of experimental animals (IACUCs) of Tianjin Institute of Environmental and Operational Medicine. The experimental procedures were carried out according to the guiding principles of animal research of the Chinese Physiological Society. No endangered or protected animal species were used in this study, and no additional harm was caused to the experimental animals.

### 4.2 Determination of serum cytokines and intestinal histopathology

Inflammatory cytokines including IL-1 β, IL-2, IL-6, IL-10, IL-12p70, TNF- α, INF- γ, and KC/GRO were measured using an electro-chemiluminescent multiplex assay from Meso Scale Discovery (MSD, Gaithersburg, MD, USA) together with a V-PLEX Proinflammatory Panel 1 Mouse Kit (MSD, catalog# K15048D) to explore the immune responses. A section of small intestine, cecum, and colon were 4% paraformaldehyde fixed and paraffin embedded, followed by standard procedures for dehydration, transparency, and wax immersion. The tissue was cut into 5-mm-thick blocks and stained using H&E before being viewed under a high-power microscope (200×, 400×). We observed the pathological changes in the intestinal samples, including intestinal mucosal continuity, goblet cell number, muscle layer injury, and epithelial edema. Intestinal tissue (1 cm) was then fixed using 4% paraformaldehyde and subjected to immunofluorescence staining, which included paraffin sectioning, antigen repair, quenching of autofluorescence, transparency, sealing, incubation with primary and secondary antibodies, re-staining with 4,6-diamino-2-phenylindole (DAPI), and nuclear blocking. Finally, a high-power microscope (200×, 400×) was used to image the immunofluorescence of the sections. Nuclei stained with DAPI appeared blue under UV excitation, and cells that were positive for CD3 and CD4 appeared red or green, respectively.^79^

### 4.3 *PBMC stimulation and recovery with metabolites* ex vivo

PBMCs were obtained using density centrifugation of diluted blood (1 mL of blood was mixed with 1 mL of cell diluent, and then added to 3 mL of cell separation solution). Cells were rinsed two times using saline and resuspended in Roswell Park Memorial Institute (RPMI) 1640 medium containing pyruvate (10 mM), L-glutamine (10 mM), and gentamicin (10 mg/mL). PBMCs were counted in a Coulter counter (Countless^TM^ 3, Invitrogen, Waltham, MA, USA) and their concentrations were adjusted to 5 × 10^6^ cells/mL. 1 × 10^6^ PBMCs in a total volume of 500 µL per well were stimulated in round-bottom 96-well plates (Corning Inc., Corning, NY, USA) with different stimulants for 8 hours at 37°C under 5% CO_2_. The respective concentrations of the stimulants were as follows: Arachidonic acid (10 μM, Macklin, Shanghai, China), Prostaglandin E2 (10 μM, SaiTong, Beijing, China), Tryptophan (500 μM, Macklin), Indole-3-acetic acid (500 μM, Macklin), which were prepared in RPMI culture medium (Procell, Wuhan, China). Supernatants were collected and stored in −80°C until used for detection.

### 4.4 Determination of the fecal bacterial community

The cetyltrimethylammonium bromide (CTAB) method was used to extract genomic DNA from fecal samples,^80^ and the purity and concentration of the DNA were detected using agarose gel electrophoresis. The diluted genomic DNA was used as the template for PCR amplification. The amplified PCR products were detected using agarose gel electrophoresis and the target bands were recovered. We then constructed a library of the PCR products employing a TruSeq DNA PCR free sample preparation kit (Illumina, San Diego, CA, USA. Quantitative real-time PCR (qPCR) and qubit fluorimetry were used to quantify the constructed library. Thereafter, the library was sequenced using the Novaseq6000 system (Illumina). FLASH (v1.2.11, http://ccb.jhu.edu/software/FLASH/) was used to splice the original data to obtain the original label.^81^ Then, Qiime’s quality control process (V2.0, http://qiime2.org/) was used to strictly filter the original data to obtain clean reads.^82^ The tag sequences were compared with the annotated species database, and chimeric sequences were removed leaving the final effective tags (https://github.com/torognes/vsearch/) .^83^ For all the samples (97%), the valid tags were clustered using Uparse software (v7.0.100, http://www.drive5.com/uparse/) .^84^ The classification information at each classification level and the number of species in each sample community were determined using the Mothur method and the SSUrRNA database (threshold 0.8 ~ 1) (http://rrna.uia.ac.be/ssu/) .^85^ Finally, the data for each sample were normalized, and the normalized data was used for α diversity and β diversity analyses.

### 4.5 Fecal and serum metabolite determination

Fecal and serum metabolites were extracted and analyzed using liquid chromatography-mass spectrometry (LC-MS).^86^ The original file (.raw) obtained from LC-MS was imported into compound discoverer 3.1 software (CD) to process the spectrum, followed by database searching to obtain qualitative and quantitative results for the metabolites. Quality control was carried out on the data to verify the reliability and accuracy of the results. The CD data processing software was used to preprocess the original data. First, the data were screened and peak aligned using the mass charge ratio and the retention time. Then, a high-resolution extracted ion flow diagram was constructed to determine the precise molecular weight of the compound according to its mass charge ratio, followed by prediction of the molecular formula based on the mass number deviation and adduct ion information. The fragment ions, collision energy, and other information for each compound were matched with data in the mzCloud database (https://www.mzcloud.org/) to identify the metabolites in the biological system. For subsequent analysis, we selected compounds with a CV (coefficient of variance) value < 30% in the QC samples as the final identification results.^87^ Next, we carried out multivariate statistical analysis of the metabolites, (partial least squares discriminant analysis (PLS-DA) and principal component analysis (PCA)) to identify the differences among the metabolites in the different groups. The relationships between the metabolites and the samples were investigated using hierarchical clustering (HCA)^88^ and metabolite correlation analysis. The identified metabolites were then annotated for their function and classification using the Kyoto Encyclopedia of Genes and genomes (KEGG) database (http://www.genome.jp/kegg/), Human Metabolome Database (HMDB) (https://hmdb.ca/), and Lipid Metabolites and Pathways Strategy (LIPID MAPS) database (https://lipidmaps.org/). Annotation of the identified metabolites using these databases allowed us to understand the functional characteristics and classification of the various metabolites.

### 4.6 Determination of intestinal antibiotic resistome in mice of different ages and sexes using metagenomics

Qubit4 (Thermo, Waltham, MA, USA) was employed to assess the concentration and purity of the extracted fecal genome. The original sequencing data was preprocessed using Readfq (V8, https://github.com/cjfields/readfq) and then SOAPdenovo software^89^ (https://github.com/aquaskyline/SOAPdenovo2) was used to assemble and analyze the preprocessed data. Scaftigs generated from the single samples and mixed assemblies that contained fragments < 500 bp were discarded.^90^ For scaftigs ≥ 500 bp,^91^ open reading frames (ORFs) were predicted using Metagenemark (V2.10, http://topaz.gatech.edu/GeneMark/. The effective data for each sample were compared with the initial gene catalog using Bowtie2 (Bowtie2.2.4), followed by calculation of the number of reads in each sample. We discarded genes with ≤ 2 reads in each sample to obtain the final gene catalog for subsequent analysis.^92^ We then calculated the abundance of each gene in each sample according to the number of reads and the gene length. Basic statistics were then derived according to the abundance of each gene in the gene catalog for each sample. The unigenes were compared with the CARD database using the resistance gene identifier (RGI) software from the CARD database (https://card.mcmaster.ca/) (RGI built-in blastp, default value ≤1e-30).^93^ Finally, the antibiotic resistance ontology (ARO) was determined and analyzed based on the comparison of the RGI values and the abundance of the unigenes.

### 4.7 Statistical analysis

Statistical analyses and mapping were carried out using GraphPad Prism V7.0 (GraphPad Inc., La Jolla, CA, USA) and RStudio (https://www.rstudio.com/), respectively. An unpaired t-test and Dunnett’s multiple comparison test were used to analyze the differences in body weight and serum cytokines of the mice in each group. The statistical significance was accepted at p < .05. In the figures, * indicates p < .05, ** indicates p < .01, and *** indicates p < .005. Qiime software (v2.0, http://qiime2.org/) was used to calculate the Chao1, Shannon and other indices in the sample complexity analysis (Alpha Diversity). Parametric and nonparametric tests were used to perform difference analysis of the α-diversity indices between the groups. For comparisons among more than two groups, we used Tukey and Wilcox tests. Next, the ggplot2 (V4.05) program in the R software package (https://www.r-project.org/) was employed to construct a PCoA diagram, heatmap, and an overview circle diagram for β-diversity analysis. Parametric and nonparametric tests were carried out to analyze differences between groups for β-diversity using the R software. We also used the Anosim, MRPP, and Adonis functions of the R vegan package. The correlation between metabolites and other detection indexes was analyzed by calculating the Pearson correlation coefficient between all metabolites and immune cytokines and the different microbiota. When the linear relationship between them increased, the positive correlation tends toward 1 and a negative correlation tends toward – 1. In addition, the significance of metabolite correlation analysis was tested statistically.^94^ A random forest model was established to distinguish disease states (random forest package). For the input features, five times cross validation (rfcv function) was used to determine the best number of identification features. Multiple hypothesis tests were adjusted using the Benjamini and Hochberg false discovery rate (FDR), and an FDR < 0.05 was considered significant.^95^ By predicting the relationship between an OTU frequency and its relative abundance, the neutral community model (NCM) was used to evaluate the contribution of random processes to the microbial community assembly. The model uses Hmisc and minpack in R code LM and other software packages were used to calculate the fitting degree and mobility of the OTUs.^96^

## Supplementary Material

Supplemental Material

## Data Availability

All data used to support the results of this study, including macrogenome data (bioproject ID: PRJNA863052) and 16S sequencing data (bioproject ID: PRJNA863739), have been uploaded to the NCBI.
