# Supplementary material for: Exogenous antibiotic resistance gene contributes to intestinal inflammation by modulating the gut microbiome and inflammatory cytokine responses in mouse
Source: Gut Microbes. 2022 Dec 27;15(1):2156764. doi: 10.1080/19490976.2022.2156764 (PMC9809935; doi:10.1080/19490976.2022.2156764)
Supplement: Supplemental Material [file KGMI_A_2156764_SM5774.zip › Supplementary file.docx]

**Supplementary Figure 1**

**
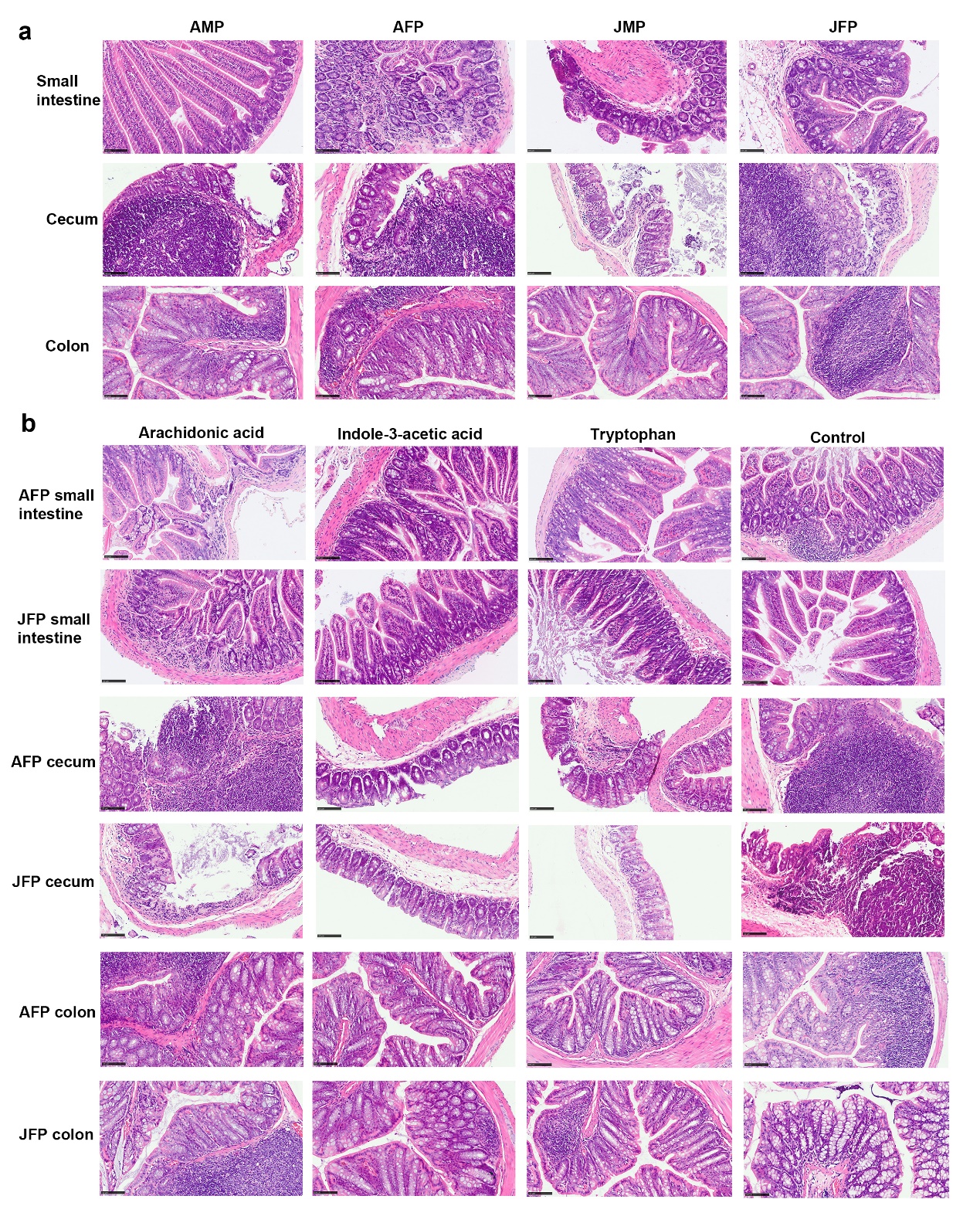
**

**Supplementary Figure 1. Pathological sections of each intestinal segment of mice stained with H&E.** Pathological changes of small intestine, cecum and colon in mice of different ages and sexes after adding plasmids (a). Pathology changes of small intestine, cecum and colon after intragastric administration of different metabolites in adult female and childhood female inflammatory mice caused by plasmids (b).

**Supplementary materials and methods**

The *mcr-1* plasmid preparation

A colistin resistance *E. col*i strain Haihe2 isolated from water samples from the Haihe River was found to carry *mcr-1* by PCR and sequencing. Total genomic DNA from Haihe2 was sequenced on a PacBio RS2 device (Pacific Biosciences, Menlo Park, CA, USA) with a 10 kb size-selected insert library. The results showed that the *mcr-1* gene was located on an IncX4 plasmid of 33.3 kb, which was assigned as pMCR-Haihe2, and no resistance determinants other than the *mcr-1* gene were found on pMCR-Haihe2. In addition, conjugation experiments were carried out in broth using azide-resistant *E. coli* strain J53 as the recipient and 2 μg/ml colistin plus 150 μg/ml sodium azide was used for selecting transconjugants. The presence of *mcr-1* in transconjugants was confirmed using PCR. The results showed that the plasmid pMCR-Haihe2 could be transferred to *E. coli* J53. The *mcr-1* plasmid was then extracted from E. coli J53 Azi^R^ conjugants and used for further experiments. Genome information of bacteria and plasmid has been uploaded to the genome in NCBI (PRJNA 904697).

Calculation formula of plasmid copy number:

$Copies/\mu l=6.02 \times{10}^{23}\times\frac{(DNA concentration (ng/\mu l)) \times{10}^{-9}}{DNA length \times660}$ (1)

The DNA length is 33307 bp, and the number of gene copies was calculated according to the formula and then diluted for intragastric administration.

**Supplementary Table 1**

| **Group** | **Inflammatory cell infiltrate** | **Epithelial changes** | **Mucosal architecture** | **Submucosal edema** | **Total score** |
| --- | --- | --- | --- | --- | --- |
| AMP-small intestine | 2 | 2 | 0 | 0 | 4 |
| AFP-small intestine | 2 | 1 | 2 | 0 | 5 |
| CMP-small intestine | 1 | 2 | 0 | 0 | 3 |
| CFP-small intestine | 3 | 0 | 0 | 0 | 3 |
| AMP-cecum | 2 | 0 | 0 | 1 | 3 |
| AFP-cecum | 3 | 1 | 1 | 1 | 5 |
| CMP-cecum | 2 | 1 | 2 | 0 | 5 |
| CFP-cecum | 3 | 1 | 2 | 0 | 6 |
| AMP-colon | 2 | 1 | 0 | 0 | 3 |
| AFP-colon | 2 | 0 | 4 | 0 | 6 |
| CMP-colon | 2 | 1 | 0 | 0 | 3 |
| CFP-colon | 3 | 1 | 0 | 0 | 4 |
| AA-AFP-small intestine | 1 | 1 | 3 | 0 | 5 |
| AA-CFP-small intestine | 2 | 1 | 2 | 0 | 5 |
| IAA-AFP-small intestine | 1 | 0 | 0 | 0 | 1 |
| IAA-CFP-small intestine | 1 | 0 | 0 | 0 | 1 |
| Trp-AFP-small intestine | 2 | 0 | 0 | 0 | 2 |
| Trp-CFP-small intestine | 1 | 0 | 0 | 0 | 1 |
| AFP-small intestine | 2 | 0 | 0 | 0 | 2 |
| CFP-small intestine | 2 | 0 | 0 | 0 | 2 |
| AA-AFP-cecumb | 3 | 1 | 2 | 1 | 7 |
| AA-CFP-cecum | 2 | 1 | 2 | 1 | 6 |
| IAA-AFP-cecum | 2 | 0 | 0 | 1 | 3 |
| IAA-CFP-cecum | 2 | 0 | 0 | 1 | 3 |
| Trp-AFP-cecum | 2 | 0 | 0 | 0 | 2 |
| TrP-CFp-cecum | 2 | 0 | 0 | 1 | 3 |
| AFP-cecum | 3 | 1 | 1 | 0 | 5 |
| CFP-cecum | 2 | 1 | 0 | 1 | 4 |
| AA-AFP-colon | 2 | 0 | 4 | 0 | 6 |
| AA-CFP-colon | 3 | 1 | 0 | 0 | 4 |
| IAA-AFP-colon | 1 | 0 | 0 | 0 | 1 |
| IAA-CFP-colon | 1 | 1 | 0 | 0 | 2 |
| Trp-AFP-colon | 1 | 0 | 0 | 0 | 1 |
| Trp-CFP-colon | 1 | 1 | 0 | 0 | 2 |
| AFP-colon | 2 | 1 | 0 | 1 | 4 |
| CFP-colon | 2 | 1 | 0 | 1 | 4 |

**Supplementary Table 2**

| **AMP** | **AMC** | **AFP** | **AFC** | **CMP** | **CMC** | **CFP** | **CFC** |
| --- | --- | --- | --- | --- | --- | --- | --- |
| Lachnospiraceae_NK4A136_group | Alistipes | Lachnospiraceae_NK4A136_group | Alistipes | Roseburia | Lachnospiraceae_NK4A136_group | Roseburia | Lachnospiraceae_NK4A136_group |
| Bacteroides | Roseburia | Bacteroides | Lactobacillus | Prevotellaceae_UCG-001 | Alistipes | Lactobacillus | Alistipes |
| Alloprevotella | Lachnospiraceae_UCG-001 | Roseburia | Mucispirillum | [Eubacterium]_xylanophilum_group | Bacteroides | Alloprevotella | Bacteroides |
| Lachnospiraceae_UCG-006 | Rikenellaceae_RC9_gut_group | Alloprevotella | [Eubacterium]_xylanophilum_group | Bilophila | Lactobacillus | Lachnospiraceae_UCG-001 | [Eubacterium]_xylanophilum_group |
| Prevotellaceae_UCG-001 | Mucispirillum | Lachnospiraceae_UCG-001 | Desulfovibrio | [Eubacterium]_siraeum_group | Alloprevotella | Lachnospiraceae_UCG-006 | Anaerotruncus |
| Desulfovibrio | [Eubacterium]_xylanophilum_group | Lachnospiraceae_UCG-006 | Colidextribacter | Anaerotruncus | Lachnospiraceae_UCG-001 | Mucispirillum | Muribaculum |
| Colidextribacter | Bilophila | Prevotellaceae_UCG-001 | Lachnoclostridium | Muribaculum | Lachnospiraceae_UCG-006 | Prevotellaceae_UCG-001 | Acetatifactor |
| Lachnoclostridium | [Eubacterium]_siraeum_group | Bilophila | Candidatus_Saccharimonas | Acetatifactor | Mucispirillum | Bilophila | Marvinbryantia |
| Oscillibacter | Blautia | [Eubacterium]_siraeum_group | Marvinbryantia | Enterorhabdus | Desulfovibrio | Colidextribacter | Acetitomaculum |
| Anaerotruncus | Acetatifactor | Blautia | Acetitomaculum | Lachnospiraceae_AC2044_group | Colidextribacter | Lachnoclostridium | |
| Muribaculum | Acetitomaculum | Anaerotruncus | Intestinimonas | | Lachnoclostridium | [Eubacterium]_siraeum_group | |
| Marvinbryantia | | Muribaculum | Butyricicoccus | | Blautia | Blautia |  |
| Enterorhabdus | | Acetatifactor | |  | Oscillibacter | Oscillibacter | |
| Parabacteroides | | Ruminococcus | |  | Marvinbryantia | Candidatus_Saccharimonas | |
| Intestinimonas | | Enterorhabdus | |  | Acetitomaculum | Ruminococcus | |
|  |  |  |  |  | Intestinimonas | Enterorhabdus | |
|  |  |  |  |  | Butyricicoccus | Intestinimonas | |
|  |  |  |  |  |  | Butyricicoccus | |

**Supplementary Table 3**

| **AMP** | **AMC** | **AFP** | **AFC** | **CMP** | **CMC** | **CFP** | **CFC** |
| --- | --- | --- | --- | --- | --- | --- | --- |
| Bacteroides | Bacteroides | Clostridium | Clostridium | Bacteroides | Bacteroides | Clostridium | Clostridium |
| Clostridium | Clostridium | Bacteroides | Eubacterium | Clostridium | Clostridium | Bacteroides | Flavonifractor |
| Alistipes | Alistipes | Eubacterium | Flavonifractor | Eubacterium | Oscillibacter | Flavonifractor | Bacteroides |
| Eubacterium | Flavonifractor | Oscillibacter | Acteroides | Oscillibacter | Flavonifractor | Oscillibacter | Eubacterium |
| Flavonifractor | Eubacterium | Flavonifractor | Oscillibacter | Alistipes | Eubacterium | Alistipes | Alistipes |
| Oscillibacter | Oscillibacter | Alistipes | Dorea | Flavonifractor | Corallococcus | Corallococcus | Oscillibacter |
| Corallococcus | Corallococcus | Acinetobacter | Corallococcus | Parabacteroides | Alistipes | Eubacterium | Corallococcus |
| Anaerotruncus | Anaerotruncus | Dorea | Alistipes | Anaerotruncus | Dorea | Roseburia | Dorea |
| Parabacteroides | Prevotella | Corallococcus | Lachnoclostridium | Corallococcus | Anaerotruncus | Prevotella | Anaerotruncus |
| Prevotella | Parabacteroides | Prevotella | Anaerotruncus | Dorea | Prevotella | Dorea | Acinetobacter |
| Dorea | Dorea | Lactobacillus | Prevotella | Acinetobacter | Parabacteroides | Anaerotruncus | Lachnoclostridium |
| Butyricicoccus | Butyrivibrio | Butyrivibrio | Butyrivibrio | Prevotella | Enterococcus | Acinetobacter | Prevotella |
| Butyrivibrio | Erysipelatoclostridium | Anaerotruncus | Blautia | Merdimonas | Acinetobacter | Butyrivibrio | Erysipelatoclostridium |
| Erysipelatoclostridium | Ruminococcus | Desulfovibrio | Lactobacillus | Lactobacillus | Lactobacillus | Erysipelatoclostridium | Butyrivibrio |
| Merdimonas | Lactobacillus | Erysipelatoclostridium | Butyricicoccus | Butyrivibrio | Roseburia | Merdimonas | Butyricicoccus |
| Lactobacillus | | Bifidobacterium | Erysipelatoclostridium | Butyricicoccus | Butyricicoccus | Blautia | Lactobacillus |
|  |  |  | Bifidobacterium | Blautia | Butyrivibrio | Lactobacillus | Roseburia |
|  |  |  | Enterococcus | | Lachnoclostridium | Ruminococcus | Ruminococcus |
|  |  |  | Eggerthella | | Mycoplasma | Clostridioides | Clostridioides |
|  |  |  | Oceanobacillus | | Desulfovibrio | Butyricicoccus | |
|  |  |  |  |  |  | Acetanaerobacterium | |
|  |  |  |  |  |  | Angelakisella | |
|  |  |  |  |  |  | Phyllobacterium | |

**Supplementary Table 4**

| **ID** | **Name** |
| --- | --- |
| B9 | Isoferulic acid |
| B10 | DL-Carnitine |
| B27 | 7-Methylguanosine |
| B33 | Phenethylamine |
| B52 | DL-Tryptophan |
| B64 | Pantothenic acid |
| B79 | Indole-3-acetic acid |
| B85 | Paracetamol |
| B92 | Hippuric acid |
| B93 | Oleoyl ethylamide |
| B106 | octadec-9-ynoic acid |
| B107 | D-(+)-Maltose |
| B109 | 3-Amino-4-methylpentanoic acid |
| B114 | 2-(acetylamino)-3-[4-(acetylamino)phenyl]acrylic acid |
| B124 | 3-Methoxy prostaglandin F1α |
| B130 | Guanidinoacetic acid |
| B137 | Spermidine |
| B149 | 6-methyl-7-nitro-2,3-dihydro-1,4-benzodioxine |
| B158 | 4-[1-(acetyloxy)prop-2-en-1-yl]-2-methoxyphenyl 2-methylpropanoate |
| B178 | Genistein 4'-O-glucuronide |
| B192 | Ureidoisobutyric Acid |
| B227 | Prostaglandin F2α-1-glyceryl ester |
| B255 | Valerophenone |
| B283 | methyl 2-[(2-acetyl-3-oxo-1-butenyl)amino]acetate |
| B287 | D-Erythrose 4-phosphate |
| B303 | Phosphocholine |
| B318 | 2-piperidinobenzoic acid |
| B341 | 2-[2-oxo-2-(pyridin-3-ylamino)ethoxy]acetic acid |
| B369 | N-α-L-Acetyl-arginine |
| B413 | Methyl 3-indolyacetate |
| B415 | Methyl-2-aminobenzoate |
| B431 | (2R)-2-[(2R,5S)-5-[(2S)-2-hydroxybutyl]oxolan-2-yl]propanoic acid |
| B438 | 4'-Methoxyacetophenone |
| B459 | Norfenefrine |
| B467 | 4-Phenyl-3-buten-2-one |
| B472 | 1-(4-hydroxyphenyl)propane-1,2-diol |
| B473 | Piperine |
| B476 | Arachidonic acid methyl ester |
| B526 | Etiocholanolone |
| B533 | gamma-Glutamylglutamic acid |
| B542 | Hexanoylcarnitine |
| B567 | N-Acetylserotonin |
| B568 | 4-(allyloxy)-1,2-dihydroquinolin-2-one |
| B572 | N-acetyl-L-ornithine |
| B607 | Dl-3-Hydroxynorvaline |
| B615 | 1,7-bis(3,4-dihydroxyphenyl)heptan-3-one |
| B640 | 4-Methyl-2-pentanone |
| B645 | 4-hydroxy-3-(3-methylbut-2-en-1-yl)benzoic acid |
| B647 | 4-Oxoretinol |
| B668 | cis-gondoic acid |
| B682 | 3-Indoleacrylic acid |
| B686 | 5-Methyl-dl-tryptophan |
| B702 | 17alpha-Ethinyl estradiol |
| B712 | 1-(2-furyl)pentane-1,4-dione |
| B751 | N-Propionylglycine |
| B760 | 7-Hydroxy-4-chromone |
| B765 | Sinapyl aldehyde |
| B770 | Prolylleucine |
| B778 | Prostaglandin E2 |
| B792 | Corey Lactone Diol |
| B797 | Hypotaurine |
| B814 | N1-(3-pyridyl)-2,3,4,5,6-pentamethylbenzene-1-sulfonamide |
| B865 | 3-Methoxytyramine |
| B869 | 2-(3,4-dimethoxyphenyl)ethanamine |
| E2 | Isotretinoin |
| E9 | N-Benzylformamide |
| E43 | Creatine |
| E47 | L-Threonine |
| E48 | Indole-3-lactic acid |
| E71 | PC (20:3/20:3) |
| E75 | 3-(3,4-dihydroxyphenyl)propanoic acid |
| E81 | Valine |
| E93 | 6-Methylquinoline |
| E107 | D-threo-Isocitric acid |
| E140 | 5-Phenylvaleric Acid |
| E144 | N-[2-chloro-6-(trifluoromethoxy)phenyl]-2,2-dimethylpropanamide |
| E166 | Prostaglandin B1 |
| E184 | Prostaglandin B2 |
| E201 | gamma-Glutamyltyrosine |
| E206 | Stearamide |
| E208 | PC (16:0/17:0) |
| E220 | PE (14:0e/22:2) |
| E224 | PC (16:2e/4:0) |
| E246 | N,N-Dimethylarginine |
| E249 | Coenzyme Q2 |
| E263 | 1,7-bis(3,4-dihydroxyphenyl)heptan-3-one |
| E272 | 3-(3,4,5-trimethoxyphenyl)propanoic acid |
| E273 | Oleoyl ethanolamide |
| E290 | 2-(2-acetyl-3,5-dihydroxyphenyl)acetic acid |
| E292 | methyl 6-{[4-(trifluoromethyl)anilino]carbonyl}nicotinate |
| E299 | Decanoylcarnitine |
| E301 | PC (16:1e/22:5) |
| E307 | PC (16:2e/22:5) |
| E322 | PC (16:0e/18:3) |
| E324 | L-Ascorbate |
| E326 | Homo-Gamma-Linolenic Acid (C20:3) |
| E349 | 3-(2-thienyl)cinnoline-4-carboxylic acid |
| E349 | 3-(2-thienyl)cinnoline-4-carboxylic acid |
| E350 | [4-(7-chloroquinolin-4-yl)piperazino](phenyl)methanone |
| E354 | 7α-Hydroxytestosterone |
| E355 | 4-(2,3-dihydro-1,4-benzodioxin-6-yl)butanoic acid |
| E371 | PC (19:2/18:3) |
| E373 | 1-(4-chlorophenyl)-2-phenylethan-1-one |
| E385 | Calcium D-Panthotenate |
| E386 | 2-{2-oxo-2H,8H,9H-furo[2,3-h]chromen-8-yl}propan-2-yl acetate |
| E413 | 2-{(3S)-1-[4-(Trifluoromethyl)benzyl]-3-pyrrolidinyl}-1H-benzimidazole |
| E417 | PE (14:1e/20:1) |
| E464 | Tetrahydrobiopterin |
| E470 | N-(4-methoxyphenethyl)-N'-(2-methylphenyl)thiourea |
| E474 | PC (19:1/18:2) |
| E475 | 4-(2,3-dihydro-1,4-benzodioxin-6-yl)-1,2-diphenylbut-2-ene-1,4-dione |
| E476 | 1-[2-(2,5-dimethyl-1H-pyrrol-1-yl)-4-nitrophenyl]-1H-imidazole |
| E500 | Deoxycytidine |
| E502 | 1-[3-(trifluoromethyl)phenyl]imidazolidine-2-thione |
| E506 | 3-(4-hydroxy-3-methoxyphenyl)propanoic acid |
| E527 | 5-Methyltetrahydrofolic acid |
| E545 | 4-Hydroxyisoleucine |
| E549 | Bilirubin |
| E555 | gamma-Glutamylleucine |
| E560 | 1-Methylhistidine |
| E563 | 5-Methoxyindole-3-Carbaldehyde |
| E572 | cis-Aconitic acid |
| E582 | Estriol |
| E584 | Prostaglandin A3 |
