# Supplementary material for: Exogenous antibiotic resistance gene contributes to intestinal inflammation by modulating the gut microbiome and inflammatory cytokine responses in mouse
Source: Gut Microbes. 2022 Dec 27;15(1):2156764. doi: 10.1080/19490976.2022.2156764 (PMC9809935; doi:10.1080/19490976.2022.2156764)
Supplement: Supplemental Material [file KGMI_A_2156764_SM5774.zip › Response.docx]

Dear Editor-in-Chief Beth McCormick, Gut Microbes

**Re: Exogenous antibiotic resistance gene contributes to intestinal inflammation by modulating the gut microbiome and inflammatory cytokine responses in mouse** (Manuscript Number: KGMI-20220830 221990620)

Thank you very much for your letter and for giving us this opportunity to modify our manuscript. The reviewers provided some comments and suggestions that were very valuable and helpful to further improve our paper. We have modified the manuscript based on the reviewer’s comments as follows:

1. The strengths and limitations of the research were added to the last paragraph of the discussion.
2. Information on the preparation and source of the resistance plasmid was added to the supplementary materials and methods.
3. Relevant results are condensed and abridged to ensure the readability of the article.
4. Incorrect words and sentences were revised.
5. Pictures and legends were modified to make them more recognizable.

Point-by-point responses are outlined below, and we have modified the manuscript according to the editor’s and reviewer’s comments (red text in the revised manuscript).

We sincerely hope that the editorial board will provide us with the opportunity to publish this paper in *Gut Microbes*.

Kind regards,

Dong Yang

**Reviewer #1**: The gut microbiota is a critical factor in many physiological and pathological processes. This research is about the contribution of the exogenous antibiotic resistance gene to intestinal inflammation by modulating the gut microbiome and inflammatory cytokine responses in mice.

**Response:** Thank you very much for your recognition of our research, and for the very meaningful suggestions, which was helpful for improving the quality of our manuscript. Our point-by-point responses to your questions are as follows:

General comments

It is a well-written article that does identify an important issue.

The subject of the paper is consistent with the scope of the journal.

The statistical analysis has been performed appropriately. The literature references are adequate.

But I suggest discussing the strengths and limitations of the research. The main Spatio-temporal similarities and differences between the mouse and human gut microbiota should be considered to translate the knowledge gained from the mouse studies to a human situation. There are some noticeable differences between mice and humans in gut microbiota composition (qualitative representation of the taxa and their quantitative contribution)

**Answer:** Thank you very much for your advice. We have considered the similarities and differences between human and mouse intestinal microbes, but we did not realize that we could discuss the limitations of the study to pave the way for transforming the knowledge gained from mouse research into human conditions. We reviewed the relevant literature and added the following discussion in the revised manuscript (**Line765-784**).

However, the limitation of this study was that there are obvious differences between the mouse and human gut microbiota. How to transfer the experimental results of mouse models to humans remains a problem that needs careful consideration in current research on the relationship between the intestinal microbiota and human diseases. There are differences in intestinal structure between mouse and human, especially with regard to the stronger fermentation ability of mouse cecum, which might affect the diversity and composition of the intestinal microbiota. Studies have shown that few bacterial species are shared between the gastrointestinal tracts of humans and mice. Through the whole genome approach, the mouse gastrointestinal bacteria catalog (MGBC) was compared with the human gastrointestinal genome (UHGG), which revealed that only 2.58% (103/3997) of species are shared between the human and mouse gut microbiotas. Of these shared species, 93.2% could be assigned a species rank by the Genome Taxonomy Database Toolkit, and 55.3% have cultured representatives within the MGBC. Although few species are shared, functions at the gene level show that up to 95% of intestinal microbial functions are shared between humans and mice. The intestinal ecosystems of the two hosts shared 84.5% of the KEGG orthology groups and 82.1% of the InterPro protein families. Therefore, mice can partly simulate the function of the human microbiota. However, to further translate the research results into the treatment of human diseases, we also plan to transplant a human microbiota into the intestines of sterile mice for research and to collect human microbiota samples for a cohort study to further reveal the impact of eARGs on humans. (**Line765-784**)

Specific Comments

1. Lines211, 725 Clarify the name of the probiotic strains involved in this study.

**Answer:** Thank you for your advice. On line 211 the probiotics mentioned include *Roseburia*, *Lactobacillus*, *Alloprevotella*, *Mucispirillum*, *Blautia*, *Oscillibacter*, *Anaerotruncus*, *Marvinbryantia*, *Ruminococcus*, *Enterorhabdus*, and *Butyricicoccus*. All of them have been listed in the manuscript, because we use 16sRNA high-throughput sequencing in this part, so only the genus level is counted. Most of these probiotics, such as *Lactobacillus*, *Oscillibacter*, *Roseburia* and *Butyricicoccus*, can decompose carbohydrates to produce small molecules that are easily absorbed by the body, such as short chain fatty acids, which are beneficial to the human health. The others are also potential probiotics, mainly responsible for fermentation in the intestine. (**Line 182-186**)

On line 725, *Roseburia*, *Eubacterium*, *Butyricicoccus*, and *Oscillibacter*. Several probiotics discussed here are extracted from the previous section (line 207). They all have the function of fermenting sugars to produce short chain fatty acids. Short chain fatty acids play an important role in maintaining the normal function of the colon and the morphology and function of colon epithelial cells. (**Line533**)

2. Lines801-803 Substantiate what is stated in these lines.

**Answer:** Thank you for your question. This part of the content is described in result section 2.4 (**Line243-248**). ARGs biomarkers are distributed in different bacteria, among which probiotics include *Lactobacillus*, *Oscillibacter*, and Bifidobacterium; opportunistic pathogens including *Alistipes*, *Prevotella*, *Clostridium*, and *Erysipelatocyclostridium*; and symbiotic bacteria including *Bacteroides*, *Enterococcus*, and *Eubacterium*. Result section 2.2 (**Line182-191**) also shows that the number of probiotics decreased and the number of pathogenic bacteria increased after the microbiota contacted eARGs. This led to a decrease in the number of beneficial metabolites related to probiotics, such as short chain fatty acids, which cannot play an active role in protecting intestinal structure and function. In addition, the number of drug-resistant pathogenic bacteria increased, and they were not easily disturbed by the external environment, thus causing continuous harm to the host, ultimately damaging host health.

3. Line826 Serotype Typhimurium capitalized, not italicized.

**Answer:** Thank you for your suggestion. We have modified the writing of the bacteria in the revision. (**Line620**)

4. Lines1004 -1005. Deeply describe the preparation of the resistance plasmid, its quantification, and intestinal persistence.

**Answer:** Thank you for your advice. In the revised supplementary materials, we added information about the preparation, source and quantification of resistance plasmid, as follows (**Supplementary materials and methods**):

The *mcr-1* plasmid preparation

A colistin resistance *E. col*i strain Haihe2 isolated from water samples from the Haihe River was found to carry *mcr-1* by PCR and sequencing. Total genomic DNA from Haihe2 was sequenced on a PacBio RS2 device (Pacific Biosciences, Menlo Park, CA, USA) with a 10 kb size-selected insert library. The results showed that the *mcr-1* gene was located on an IncX4 plasmid of 33.3 kb, which was assigned as pMCR-Haihe2, and no resistance determinants other than the *mcr-1* gene were found on pMCR-Haihe2. In addition, conjugation experiments were carried out in broth using azide-resistant *E. coli* strain J53 as the recipient and 2 μg/ml colistin plus 150 μg/ml sodium azide was used for selecting transconjugants. The presence of *mcr-1* in transconjugants was confirmed using PCR. The results showed that the plasmid pMCR-Haihe2 could be transferred to *E. coli* J53. The *mcr-1* plasmid was then extracted from E. coli J53 Azi^R^ conjugants and used for further experiments. Genome information of bacteria and plasmid has been uploaded to the genome in NCBI (PRJNA 904697).

Calculation formula of plasmid copy number:

$Copies/\mu l=6.02 \times{10}^{23}\times\frac{(DNA concentration (ng/\mu l)) \times{10}^{-9}}{DNA length \times660}$ (1)

The DNA length is 33307 bp, and the number of gene copies was calculated according to the formula and then diluted for intragastric administration.

The content of the plasmid harboring *mcr-1* in feces was continuously monitored. The results showed that large amounts of the plasmid were excreted with feces. However, we observed that *mcr-1* correlated positively with inflammatory cytokine levels (**Fig. 4j**), indicating that it had invaded into gut microbial community.

**Reviewer #2**: The authors have done a wonderful work by using different methods, assays, and approaches to investigate the effects of eARGs on the microbiome assembly, host cytokines, and metabolites in mice of different ages and either sex. The article is generally well written and very detailed, with minor grammatical corrections, some of which I have pointed out in the attached document.

**Answer:** Thank you very much for your recognition of our research, and for these meaningful suggestions, which have helped to improve the quality of our manuscript.

Besides my comments in the attached PDF document, the authors must look at cutting down on the size of the article; it's too long, which makes it cumbersome to read. Please improve on the figures and the figure legends as they are illegible.

**Answer:** Thank you very much for your advice. Our point-by-point responses to your questions are as follows:

1. The comments in the attached PDF document.

**Answer:** Thank you very much for your careful reading and making so many detailed modifications to our manuscript. We have replaced and deleted relevant words, changed some abbreviations and genes into correct writing, and modified some sentences and paragraphs. The modified parts are replaced with red.

(1) The expressed microbiota is wrongly written as “microbiome” and has been replaced with “microbiota”. (**Line17, 41, 50, 63, 72, 92**)

(2) Related expressions in the text are wrongly written as “linked” and have been replaced with “associated”. (**Line14, 26, 44, 78**)

(3) We have replaced “How variations in the composition and metabolomics function of the gut microbiota led to cytokine responses to eARG stimulation in mice of different age and sex were investigated” in the abstract with “How eARG-stimulated variations in the composition and metabolomic function of the gut microbiota led to cytokine responses in mice of different age and sex were investigated” as you suggested. (**Line22-24**)

(4) We have changed “The human gut microbiota is composed of densely colonized microorganisms” in the introduction to the correct expression “The human gut microbiota is densely colonized by micro-organisms”. (**Line40**)

(5) The whole process of these antibiotic resistance genes is as follows. However, in relevant articles, antibiotic resistance genes are generally in the form of abbreviations. Because we only mention them once in the introduction of the manuscript, we use the abbreviations that everyone agrees with. But there are also some problems in our writing. We have modified the revision to *bla*_NDM_, *bla*_KPC_, *mcr-1, PMQR, optrA* (**Line 67**).

New Delhi metallo-β-lactamase (NDM)

Klebsiella pneumoniae carbapenemase (KPC)

Plasmid mediated-quinolone resistance (PMQR)

Plasmid mediated-colistin resistance gene (mcr)

Oxazolidinone/phenicol resistance gene (optrA)

(6)The full name has been added where the abbreviation is first used in the text. (**Line 46, 112-114, 171, 415, 773-774, 1207**)

(7) The relevant reference has been added to “Therefore, eARGs might directly enter the human intestine through drinking water and foods, and then interact with the intestinal microbiota. Many studies have shown that ARGs can transfer into the intestinal microbiota”. (**Line72**)

(8) Some inappropriate words have been modified according to your suggestions in the revision. (**Line 32, 43, 49, 69, 92, 103, 112, 128**)

2. The authors must look at cutting down on the size of the article; it's too long, which makes it cumbersome to read.

**Response:** Thank you very much for your advice, which is crucial to the improvement of the quality of our article. We have cut and condensed the relevant paragraphs of the results according to your suggestion, and presented them mainly in the form of figures and supplementary tables in the revision, to avoid redundant description, which is not conducive to reading. (**Line 188-189** (Fig. 2h-2k, Table S2); **Line 225-232**; **Line238-243** (Fig. 4d-4g, Table S3))

3. Please improve on the figures and the figure legends as they are illegible.

**Response:** We are very sorry that the relevant icons in the pictures and legends cannot correspond to each other due to our carelessness, which makes them difficult to distinguish. In addition, we had written the legend of Figure 5 and Figure 6 in the wrong place. We have modified the relevant figures and legends in the revision. (**Line1164-1270**)
